# Soil selenium activator mediates selenium form transformation and affects soil properties and microbial communities

**DOI:** 10.3389/fmicb.2026.1715630

**Published:** 2026-04-15

**Authors:** Xiangmei Zhao, Zhizong Liu, Yonglin Wu, Tao He, Liu Gao, Li Bao, Hongyin Zhou, Sheng Wang, Naiming Zhang

**Affiliations:** 1 College of Resources and Environment, Yunnan Agricultural University, Kunming, China; 2 Yunnan Hanzhe Science and Technology Co., Ltd., Kunming, China; 3 Yunnan Key Laboratory of Gastrodia and Fungi Symbiotic Biology, Zhaotong University, Zhaotong, Yunnan, China

**Keywords:** bacterial diversity, bioavailability, fungal diversity, selenium forms, soil selenium activator

## Abstract

**Introduction:**

Addressing global crop selenium (Se) deficiency requires novel strategies that enhance the bioavailability of native soil Se while minimizing environmental impact. This study introduced a novel organic-inorganic composite activator to achieve this goal.

**Methods:**

The composite activator was formulated by combining wood vinegar (WV), biochemical fulvic acid (BFA), and alginic acid (AA) with phosphorus tailings (PT), thereby avoiding the resource consumption and pollution risks associated with external Se input. Through orthogonal experimental design integrated with high-throughput sequencing, correlation analysis, co-occurrence network modeling, and functional prediction, this study systematically elucidated how the composite activator synergistically regulates Se speciation transformation, soil properties, and microbial community structure.

**Results:**

The optimized Z8 formulation yielded the following results: (1) Compared to the control (CK) (25.2 μg/kg), the bioavailable Se content under the Z8 treatment (39.2 μg/kg) significantly increased by 56%; and (2) The co-application of PT (containing 8.63% P) and organic acids increased soil available phosphorus (AP) by 21.2% after 60 days.

**Discussion:**

In conclusion, this study proposes a sustainable strategy of amending soil with organic acid-activated PT, which synergistically improves soil Se and P fertility while offering a new pathway for the resource utilization of industrial waste.

## 1 Introduction

The low bioavailability of soil Se, primarily determined by its chemical speciation, significantly limits its enrichment in crops (Mora et al., 2015; Ros et al., 2016). Specifically, the Soluble Se (SOL-Se) and exchangeable Se (EX-Se) fractions exhibit the highest bioavailability and can be directly absorbed by plants (Di Tullo et al., 2016). Se bound to carbonates (CARB-Se) and Fe-Mn oxides (FMO-Se) can be transformed into soluble and exchangeable forms via dissolution and desorption mechanisms. While Humic Acid-bound Se (HA-Se) and Organic Matter-bound Se (SOM-Se) are categorized as organic Se, they can slowly release soluble organic and inorganic Se, making them potentially available for plant uptake (Liao et al., 2024). Conversely, residual Se (RES-Se), though highly stable and exhibiting negligible bioavailability, serves as an important long-term Se reservoir in soil (Qu et al., 2023). Conventional strategies for enhancing Se content often rely on exogenous Se supplementation, such as foliar spraying or soil fertilization (Peng et al., 2017). However, the utilization efficiency of soil-applied Se is often below 5–10%, with the remainder accumulating in the soil or being lost through volatilization, leaching, or runoff, thereby posing potential risks to ecosystems (Li et al., 2017). Therefore, developing strategies to convert stable Se forms into bioavailable is crucial to improve its bioavailability (Liu et al., 2021).

Organic compound enhance Se bioavailability and facilitate Se speciation transformation by modifying fundamental soil properties (Qu et al., 2023; Qi et al., 2025). Studies have shown that these activators increase the content of SOL-Se, EX-Se, and fulvic acid-bound Se (FA-Se) (Wang et al., 2018). Soil organic (SOM) matter plays a key role in regulating Se dynamics. Adding organic complexing activators to Se-enriched soils Promotes Se retention and reduces the loss of SOL-Se (Wang et al., 2019). Furthermore, organic amendments increase dissolved organic matter in the short term, this highly reactive fraction is readily decomposed by microorganisms, consequently stimulating the release of immobilized Se (Moreno-Jiménez et al., 2013; Supriatin et al., 2015)Soil pH also influences Se bioavailability by affecting surface charge, adsorption-desorption behavior, and redox stability; specifically, lower pH often promotes Se immobilization due to enhanced adsorption onto positively charged soil minerals (Eswayah et al., 2016). Beyond physicochemical properties, Organic activators can alter the structure and function of soil bacterial communities, further enhancing Se bioavailability (Gong et al., 2024). Microorganisms are crucial for regulating Se availability and driving its biogeochemical cycling, ultimately influencing plant uptake (Li et al., 2020). Various microbial genes and proteins participate in Se methylation and transformation (Pinel-Cabello et al., 2021). Furthermore, Se influences microbial metabolic activities, which helps maintain biodiversity and ecosystem stability (Zhang M. et al., 2019). Microorganisms mediate Se oxidation and reduction, altering its speciation and availability (Wang Z. et al., 2022). Specifically, some mediate the conversion of Se from higher oxidation states to forms more readily assimilated by plants, while others oxidize organic Se to inorganic forms, Mechanisms such as extracellular phosphatase secretion and redox reactions collectively accelerate the Se cycle (Kushwaha et al., 2022; Fischer et al., 2020)

Soil microbial communities are a crucial component of soil ecosystems, with their structure and function heavily influenced by environmental factors (Li et al., 2020). During Se transformation, microorganisms catalyze the conversion of different Se valence states by encoding specific reductases or oxidases (Wang D. et al., 2022). However, the mechanisms underlying composite activators, which may include components such as DOM, wood vinegar, alginate, biochar, or specific microbial inoculants, are more complex. Specifically, DOM provides labile organic carbon, significantly promoting the metabolic proliferation and activity of selenium-reducing bacteria (e.g., *Desulfobulbus* and *Pseudomonas*) (Han et al., 2020). Through its redox properties, DOM can also directly or indirectly participate in bioreduction by acting as an electron donor (Qiao et al., 2019). Wood vinegar, rich in phenolic and organic acid compounds, selectively stimulates functional taxa (e.g., Pseudomonas and Bacillus) by optimizing soil conditions, such as pH and carbon availability (Zhang F. et al., 2019; Fan et al., 2022). Additionally, it enhances the activity of humus-degrading enzymes (e.g., β-glucosidase), increasing soil DOM content and further fueling Se transformation (Zheng et al., 2025). Similarly, alginate and its degradation products (e.g., alginate oligosaccharides) serve as carbon sources to stimulate specific metabolic activities (Xing et al., 2020). While Microbial inoculants directly introduce functional strains to boost biological activity (Cheng et al., 2021). These alterations create a complex feedback loop where changes in microbial communities and Se speciation subsequently modify soil physicochemical properties (Fellowes et al., 2013; Deng et al., 2025). Despite this, the causal mechanisms linking composite activators, specific microbial functions, Se transformation pathways, and soil properties remain unclear.

Unraveling how interactions within microbial communities (e.g., among different bacterial or fungal taxa) and between microorganisms and the plant rhizosphere (e.g., recruitment of microorganisms by rhizosphere secretions) synergistically promote or inhibit Se transformation under the influence of composite activators is an area of active research (Wang et al., 2024; Deng et al., 2025). Existing studies indicate that composite activators can regulate Se transformation by altering microbial community structure, yet the underlying mechanisms governing the causal relationships among composite activators, specific microbial communities/functions, key Se transformation pathways, and soil properties remain unclear.

While organic acids like humic acid and Wood vinegar effectively activate soil Se (Khalid et al., 2022). their application often lowers soil pH, exacerbating acidification (Najafi-Ghiri et al., 2022), and fails to supply essential nutrients like phosphorus. PT a solid waste from wet-process phosphoric acid production, offer a potential resource solution (Wu et al., 2022). This study develops a composite activator by combining organic acids with PT. This approach aims to not enhance Se bioavailability but also leverage organic acids to solubilize phosphorus from the tailings, thereby increasing soil AP. Crucially, the alkaline nature of the tailings helps neutralize the acidity introduced by organic acids, an orthogonal experimental design was employed to optimize the formulation for improving soil properties, regulating Se transformation, and shaping microbial community structure.

## 2 Materials and methods

### 2.1 Test material

Soil samples were collected from the topsoil layer (0–20 cm) of farmland in Yuxi, Yunnan, China. The samples were naturally air-dried in the laboratory, ground and passed through a 2-mm sieve prior to use. The basic physicochemical properties of the soil were as follows: SOM, 50.83 g/kg; pH, 7.02; alkali-hydrolysable nitrogen (AN), 132.75 mg/kg; AP, 162.17 mg/kg; available potassium (AK), 312.50 mg/kg; and total Se, 0.43 mg/kg, and the absolute concentrations and percentage changes of various selenium species are shown in Table 1. To achieve synergistic activation of native soil Se, we selected four functionally complementary materials: (1) WV, which modifies Se speciation via rapid soil acidification induced by its organic acids (Xu et al., 2024); (2) BFA, which facilitates the release of fixed Se through competitive desorption and chelation (Urík et al., 2018) ; (3) AA, which forms soluble complexes with Se via its abundant anionic functional groups, thereby enhancing solubility (Yang et al., 2011; Xu et al., 2024); and (4) PT, which selenate through the competitive adsorption of phosphate ions for binding sites (de la Mora et al., 2008). Collectively, these components establish a coordinated activation system operating via “acid dissolution-reduction-complexation-competitive adsorption” pathways. The materials were included obtained from the following sources: WV from the Yunnan Engineering Research Center for Soil Fertilization and Pollution Remediation; BFA and AA from Wuzhoufeng Agricultural Technology Co., Ltd.; and PT from Yunnan Yuntianhua Co., Ltd.

**TABLE 1:** Absolute concentrations (Unit: mg kg^−1^) and percentage changes of various Se species.

	SOL-Se		EX-Se		CARB-Se		HA-Se		FMO-Se		SOM-Se		RES-Se	
	Concen- tration (mg kg^−1^)	Percen- tage (%)	Concen- tration (mg kg^−1^)	Percen- tage (%)	Concen- tration (mg kg^−1^)	Percen- tage (%)	Concen- tration (mg kg^−1^)	Percen- tage (%)	Concen- tration (mg kg^−1^)	Percen- tage (%)	Concen- tration (mg kg^−1^)	Percen- tage (%)	Concen- tration (mg kg^−1^)	Percen- tage (%)
CK	0.012 ± 0.07	2.76	0.013 ± 1.4	3.47	0.016 ± 0.16	3.85	0.137 ± 1.5	31.62	0.011 ± 0.78	2.33	0.118 ± 0.39	27.56	0.122 ± 0.19	28.42
Z1	0.013 ± 1.48	4.14	0.018 ± 2.01	4.68	0.053 ± 3.33	13.61	0.104 ± 3.43	22.88	0.011 ± 0.56	2.32	0.109 ± 2.5	24.65	0.120 ± 0.92	27.72
Z2	0.017 ± 0.94	3.77	0.017 ± 1.64	4.33	0.056 ± 3.88	13.92	0.1054 ± 3.55	22.92	0.011 ± 0.41	2.54	0.110 ± 4.21	24.84	0.119 ± 0.69	27.67
Z3	0.017 ± 0.99	3.81	0.02 ± 2.58	4.92	0.054 ± 6.2	13.70	0.106 ± 2.51	23.18	0.010 ± 0.28	2.37	0.108 ± 3.38	24.26	0.120 ± 1.09	27.75
Z4	0.016 ± 1.54	3.68	0.017 ± 1.3	4.39	0.057 ± 3.42	13.92	0.103 ± 5.11	23.16	0.011 ± 0.51	2.46	0.109 ± 2.83	24.69	0.120 ± 1.47	27.71
Z5	0.016 ± 0.8	3.85	0.019 ± 2.71	4.85	0.059 ± 2.84	14.42	0.101 ± 7.24	22.00	0.011 ± 0.20	2.41	0.109 ± 4.41	24.73	0.121 ± 1.31	27.75
Z6	0.016 ± 0.8	3.90	0.019 ± 2.47	5.06	0.058 ± 2.24	14.05	0.103 ± 3.62	22.28	0.010 ± 0.65	2.33	0.109 ± 4.53	24.45	0.121 ± 0.93	27.93
Z7	0.018 ± 2.14	3.81	0.019 ± 2.01	4.82	0.057 ± 4.42	14.43	0.102 ± 3.68	22.35	0.010 ± 0.18	2.34	0.109 ± 2.92	24.73	0.120 ± 1.49	27.52
Z8	0.019 ± 1.27	4.40	0.02 ± 3.63	5.42	0.062 ± 3.88	15.13	0.101 ± 5.84	21.20	0.10 ± 0.49	2.19	0.105 ± 5.11	23.71	0.121 ± 0.48	27.95
Z9	0.017 ± 3.03	3.67	0.02 ± 3.98	5.28	0.058 ± 3.04	14.25	0.104 ± 4.58	22.65	0.10 ± 0.72	2.20	0.108 ± 3.74	24.23	0.120 ± 1.19	27.71

### 2.2 Experimental design

An L9(3^4^) orthogonal experimental was employed to optimize the formulation of the composite activator for enhancing soil Se content. Four key factors-BFA, AA, WV, and PT, were evaluated at three levels (Table 2). The experiment consisted of ten treatments: nine orthogonal combinations (Z1-Z9) and a blank control (CK) without additives, with each treatment replicated three times. The values in Table 2 represent the mass fraction of each component relative to the total application rate and dosages were calculated according to the orthogonal layout (Table 3). The materials were weighed and thoroughly mixed with 200 g of the tested soil, then transferred to 40 mm × 40 mm culture boxes. soil moisture content was maintained at 75 and 85% of field capacity using the gravimetric method, and incubation was conducted at 25°C. Soil samples (10 g) were collected at 7 d, 15, 30, and 60 days to determine SOM, pH, AP, electrical conductivity (EC), cation exchange capacity (CEC), and Se speciation. At 60 days, the remaining soil was collected for high-throughput sequencing analysis.

**TABLE 2:** Factors and levels for the orthogonal experimental design of the soil Se composite activator.

/	HF	HZ	MC	KZ
1	20%	0%	50%	30%
2	25%	10%	40%	25%
3	30%	20%	30%	20%

**TABLE 3:** Orthogonal experimental design and absolute dosage of components for the soil se composite activator.

Series no	HF	HZ	MC	KZ
CK	0	0	0	0
Z1	1.6	0	4.0	2.4
Z2	1.6	0.8	2.4	2.0
Z3	1.6	1.6	3.2	1.6
Z4	2.0	0	2.4	1.6
Z5	2.0	0.8	3.2	2.4
Z6	2.0	1.6	4.0	2.0
Z7	2.4	0	3.2	2.0
Z8	2.4	0.8	4.0	1.6
Z9	2.4	1.6	2.4	2.4

Unit: g per 200 g soil.

Orthogonal experimental design is primarily employed to screen key factors rather than to construct precise mathematical models. Unlike Response Surface Methodology, orthogonal design involves fewer experimental points, making it efficient for optimization but less suitable for fitting high-precision non-linear regression equations. In this study, we adopted an L9(3^4^) orthogonal design to systematically analyze the main effects of four factors (WV, BFA, AA, PT) at three levels using a limited number of experimental runs (9 orthogonal treatments plus 1 control). This approach enabled the scientific identification of the optimal formulation (Z8). Furthermore, subsequent field trials will be conducted to validate the efficacy of this optimized composite activator.

### 2.3 Measurement items and analytical methods

Soil Se fractions were determined according the Technical Requirements for Sample Analysis of Eco-geochemical Evaluation (DD 2005-03). Bioavailable Se was calculated as the sum of the SOL-Se and EX-Se, which were sequentially extracted using deionized water and 1 mol/L MgCl_2_, respectively, and quantified via atomic fluorescence spectrometry. Soil pH and EC were measured in a 1:25 (w/v) soil/0.01 M CaCl_2_ suspension, while cation CEC was determined spectrophotometrically using the hexaminecobalt trichloride method (Liu et al., 2022). AP was determined NaHCO_3_ extraction followed by Molybdenum-antimony anti-spectrophotometry (Zhou et al., 2019), SOM was determined using the potassium dichromate oxidation-spectrophotometric method. Briefly, soil organic carbon was oxidized by a K_2_Cr_2_O_7_–H_2_SO_4_ solution under external heating. And The concentration of the reduced Cr^3+^ was measured at 590 nm using glucose as the standard for the calibration curve (Liu et al., 2022).

Total genomic DNA was extracted from the samples using the DNeasy^®^ PowerSoil^®^ Pro Kit (QIAGEN, United States). DNA integrity and purity were verified via 1% agarose gel electrophoresis and quantified using a NanoDrop 2000 spectrophotometer. The V3-V4 region of the bacterial 16S rRNA gene was amplified using primers 338F (5′-ACTCCTACGGGAGGCAGCAG-3′) and 806R (5′-GGACTACHVGGGTWTCTAAT-3′), while the fungal ITS1 region was amplified using primers ITS1F (5′-CTTGGTCATTTAGAGGAAGTAA-3′) and ITS2R (5′-GCTGCGTTCTTCATCGATGC-3′) (Wen et al., 2020; Chen et al., 2022). Primers were synthesized by Majorbio Bio-Pharm Technology Co., Ltd. (Shanghai, China). The polymerase chain reaction was performed in a 20 μL reaction mixture containing 4 μL of 5 × FastPfu Buffer, 2 μL of dNTPs (2.5 mmol/L), 0.8 μL of each primer (5 μmol/L), 0.4 μL of TransStart^®^ FastPfu DNA Polymerase, 10 ng of DNA template, and dd H_2_O. The thermal cycling conditions were as follows: initial denaturation at 95 °C for 3 min; followed by 30 cycles of denaturation at 95 °C for 30 s, annealing at 55 °C for 30 s, and extension at 72 °C for 45 s; and a final extension at 72 °C for 10 min. The reaction was held at 10 °C. PCR products were purified from 2% agarose gels using the AxyPrep DNA Gel Extraction Kit (Axygen Biosciences, United States) and quantified using a Quantus Fluorometer (Promega, United States). Purified amplicons were pooled and sequenced on the Illumina MiSeq PE300 platform by Majorbio Bio-Pharm Technology Co., Ltd. Raw reads were deposited in the NCBI Sequence Read Archive (SRA) database (Accession No. PRJNA1355499).

### 2.4 Data analysis

Raw sequence data were quality-filtered using Fastp (Chen et al., 2018) and merged using FLASH. Operational taxonomic unit (OTUs) were clustered at a 97% similarity threshold using UPARSE, with simultaneous chimera removal (Stackebrandt and Goebel, 1994). Taxonomic annotation was performed using the RDP Classifier (confidence threshold: 70%) against the SILVA 16S rRNA and UNITE ITS databases (Wang et al., 2007). Bioinformatic analyses were conducted on the Majorbio Cloud Platform (Majorbio Bio-Pharm Technology Co., Ltd., Shanghai, China). Alpha diversity indices were calculated using Mothur (v1.30.2), and intergroup differences were assessed using *t*-tests. Beta diversity was evaluated via non-metric multidimensional scaling based on Bray-Curtis distances, coupled with Analysis of Similarities using 999 permutations. Taxonomic differences at the phylum level were analyzed using Wilcoxon rank-sum tests with two-tailed confidence intervals (calculated via Bootstrap) and False Discovery Rate correction. Data were initially processed using Microsoft Excel 2021 for mean and standard deviation calculations. Statistical analyses, including one-way ANOVA with Duncan’s *post-hoc* test and Pearson’s correlation analysis, were performed using IBM SPSS Statistics 26 (*P* < 0.05). Microbial functions were predicted using FUNGuild 1.0, PICRUSt2 2.2.0, and FAPROTAX. Co-occurrence networks were constructed in R 4.3.3 using the psych, Hmisc, and igraph packages and visualized using Gephi 0.9.7. Figures were generated using Origin 2021 and further refined using the Hiplot platform, the Lianchuan Bio-Cloud platform, and Adobe Illustrator 2022.

## 3 Results

### 3.1 The effect of soil Se activators on the basic soil properties

**FIGURE 1:**
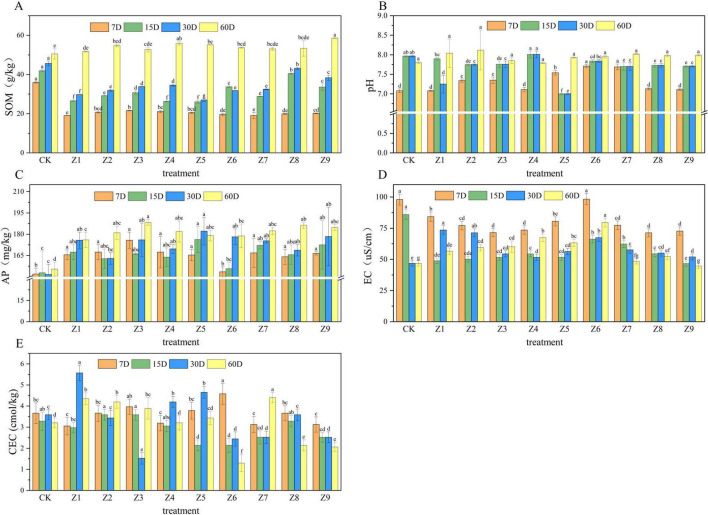
Effects of the composite activator on basic soil properties: **(A)** SOM, **(B)** pH, **(C)** AP, **(D)** EC, and **(E)** CEC. For each sampling time, different lowercase letters above the bars indicate significant differences among treatments (*P* < 0.05, Duncan’s multiple range test). This convention applies to all Figures.

Figure 1 illustrates the temporal dynamics of basic soil properties following the application of the composite Se activator. SOM exhibited a biphasic trend: a slow initial increase followed by a rapid accumulation Notably, Which SOM levels in all treatment groups were significantly lower than the CK during the early stages (7–30 days), levels in the Z2, Z4, Z5, Z6, and Z9 groups surged by 60 d, significantly surpassing the CK and ranging from 14.55 to 38.37 g/kg (Figure 1A). Soil pH gradually increased in the Z2, Z3, Z6, Z7, Z8, and Z9 groups. Specifically, pH values in Z2, Z3, Z5, and Z7 were significantly higher than CK form 7 to 30 days; However, by 60 days, pH stabilized across all groups, showing no significant intergroup differences. AP the Z5, Z7, and Z9 groups consistently maintained significantly higher levels compared to CK throughout the incubation period (Figure 1C). Electrical conductivity (EC) in groups Z3, Z5, and Z7 showed a significant decline from 7 to 15 days, followed by a subsequent increase, whereas no discernible temporal pattern was observed For CEC across treatment (Figures 1D,E).

### 3.2 The effect of soil Se activators on the Se soil fractions

**FIGURE 2:**
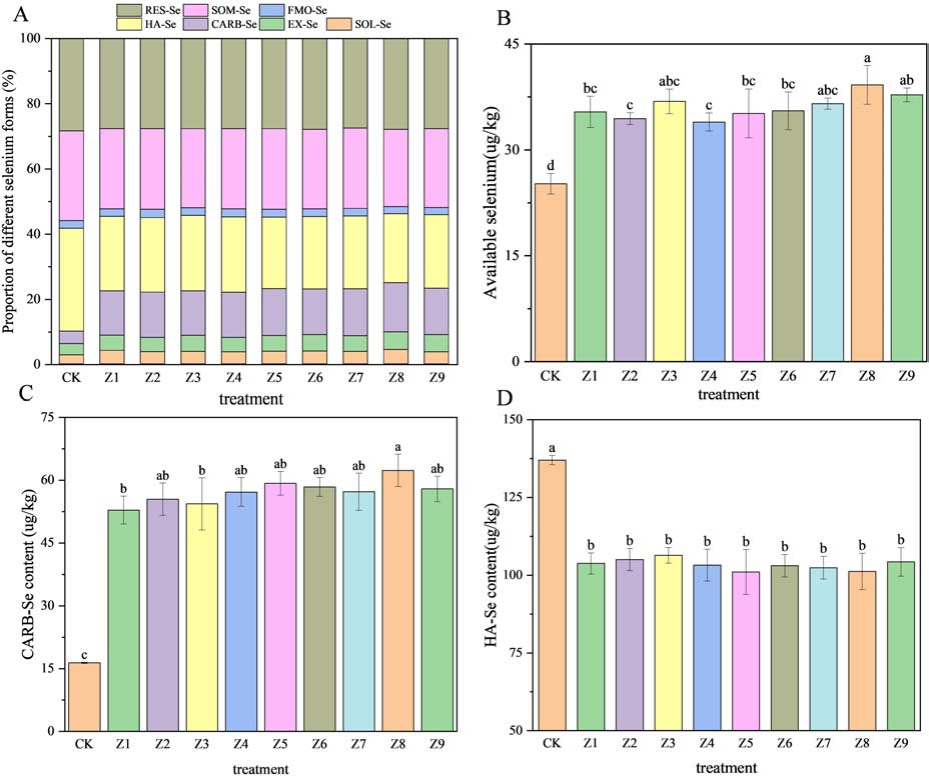
Changes in soil Se fractions. **(A)** Percentage distribution of Se forms. **(B)** Content of bioavailable Se (SOL-Se + EX-Se). **(C)** Content of carbonate-bound Se. **(D)** Content of humic acid-bound Se.

Figure 2 illustrates the distribution of Se fraction in the soil following different treatments. Se was primarily distributed as HA-Se (21.2–31.6%), SOM-Se (23.7–27.6%), and RES-Se (27.5-28.4%), with CARB-Se accounting for 3.9–15.1%. Compared to the CK (25.2 μg/kg), the application of composite activators (Z1–Z9) significantly enhanced bioavailable Se content. Specifically, increases ranged from 35% in the least effective treatment (Z4, 33.93 μg/kg) to 56% in the most effective treatment (Z8, 39.2 μg/kg). Regarding specific fractions, CARB-Se content was significantly higher in all treated soils than in CK, showing a maximum increase of 11.3%. Conversely, HA-Se content was significantly reduced in all treatment groups, decreasing by up to 10.4% relative to CK. No significant differences were observed in RES-Se levels across the treatment groups.

### 3.3 The effect of soil Se activators on soil microorganisms

#### 3.3.1 Microbial composition of the soil

**FIGURE 3:**
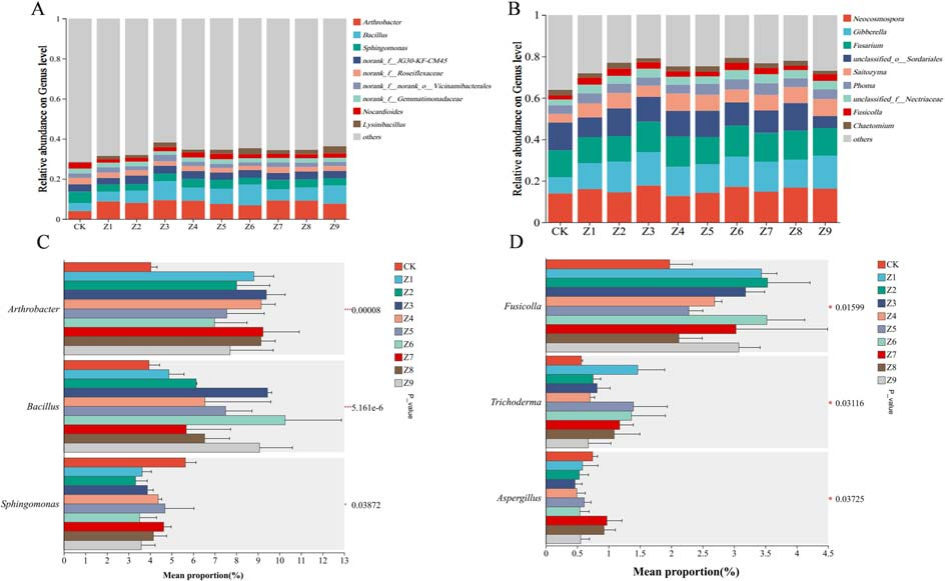
Comparative analysis of soil microbial community composition. **(A)** Bar plot of bacterial community composition at the genus level. **(B)** Bar plot of fungal community composition at the genus level. **(C)** Multi-comparison plot of bacterial genus relative abundance. **(D)** Multi-comparison plot of fungal genus relative abundance. Panels C and D are annotated with significance values (*P*-values, where asterisks indicate significant differences), directly illustrating the significance of differences in the same genus across different groups. The Y-axis represents genus names at the taxonomic level, the X-axis represents the mean relative abundance of genus in different groups, and bars of different colors represent different groups. The values on the far right indicate *P*-values, with significance levels denoted as: * for 0.01 < *P* ≤ 0.05, ** for 0.001 < *P* ≤ 0.01, and *** for *P* ≤ 0.001.

As illustrated in Figure 3, the application of various compound activators induced significant shifts in the soil microbial community structure relative to the CK. At the bacterial genus level, the relative abundances of *Arthrobacter* and *Bacillus* were significantly enhanced, whereas *Sphingomonas* declined markedly regarding fungi, the relative abundances of *Fusicolla*, *Trichoderma*, and *Aspergillus* exhibited notable increases. Overall, the activator treatments led to a general trend characterized by a decrease in total bacterial abundance and a concurrent increase in total fungal abundance. Specifically, the relative abundance of *Arthrobacter* was significantly higher in treatments Z3, Z8, and Z9, while *Bacillus* was significantly enriched in Z3, Z6, and Z9. regarding fungal taxa, *Fusicolla* was more abundant in Z1, Z2, and Z6; *Trichoderma* increased significantly in Z7 and Z8; and *Aspergillus* levels were elevated in Z1, Z5, and Z6.

#### 3.3.2 Analysis on the diversity of soil microorganisms

**FIGURE 4:**
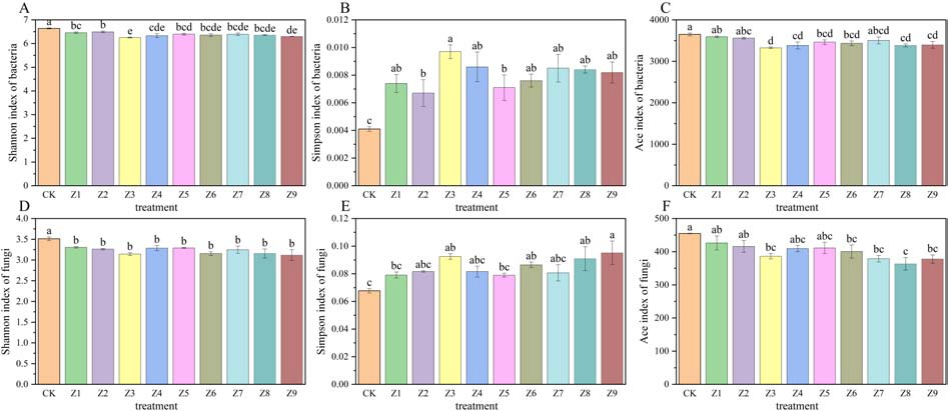
Soil microbial alpha diversity indices. **(A–C)** Bacterial diversity indices (Shannon, Simpson, Ace) among different treatments at 60 days. **(D–F)** Fungal diversity indices (Shannon, Simpson, Ace) among different treatments at 60 days. Asterisks indicate significant differences compared to the control group (CK).

The Shannon, Simpson, and Ace indices were calculated to assess the diversity and richness of soil microbial communities. As shown in Figure 4, the application of soil activators generally reduced the diversity and richness of both bacterial and fungal communities. Notably, fungi were sensitive to all treatments, whereas bacteria in specific groups (Z1, Z2) maintained diversity levels comparable to the CK. Specifically, the bacterial Shannon index was highest in CK and lowest in Z3, the latter being significantly lower than that in other treatments. Conversely, the bacterial Simpson index exhibited the opposite trend—lowest value in CK and highest in Z3—indicating that Z3 treatment significantly increased bacterial dominance while reducing evenness. The bacterial ACE index remained high in CK, Z1, and Z2, but was significantly reduced in Z3, Z4, Z8, and Z9. Regarding fungi, the Shannon index significantly decreased across all treatment groups, showing consistent inhibitory trends. The fungal Simpson index was lowest in CK and relatively higher in Z3, Z8, and Z9. The fungal ACE index was highest in CK and lowest in Z8, with richness generally lower in all treatment groups compared to CK. In summary, The Z3 treatment induced a “reduced diversity and increased dominance” pattern in both bacterial and fungal communities, potentially leading to simplified community structure. Treatments Z1 and Z2 had minor effects on bacterial diversity, whereas Z8 and Z9 exerted strong inhibitory effects on the richness of both kingdoms. These results indicate that soil activators generally reduces microbial diversity, with fungi demonstrating higher sensitivity than bacteria, while retained control-level diversity under specific treatments (Z1, Z2).

#### 3.3.3 Characterization of soil microbial networks

**FIGURE 5:**
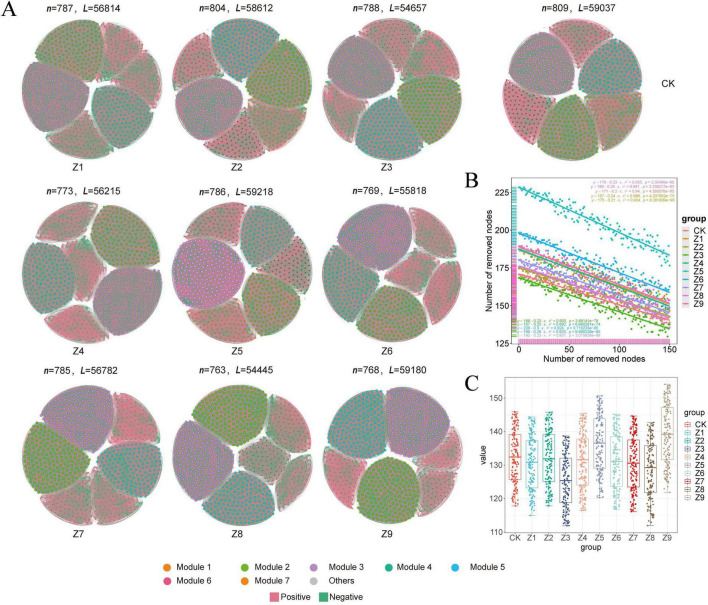
Bacterial co-occurrence network and topological properties. **(A)** Individual nodes represent individual OTUs, with their size proportional to the node degree and colors indicating modules. Edges represent significant Spearman correlations (| *R*| > 0.7, *P* < 0.05). Red lines indicate positive correlations, while green lines indicate negative correlations. **(B)** Network stability testing, where different colors represent different treatments, and the equation describes the change in natural connectivity. **(C)** Community complexity analysis, where different letters indicate statistically significant differences determined by *t*-test.

**FIGURE 6:**
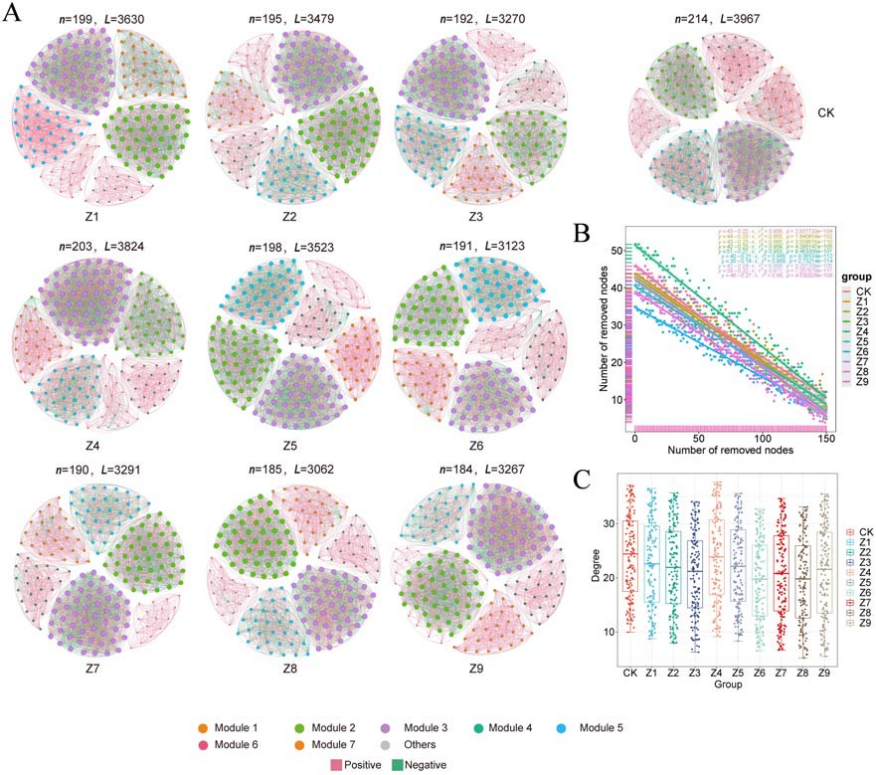
Fungal co-occurrence network and topological properties. **(A)** Individual nodes represent individual OTUs, with their size proportional to the node degree and colors indicating modules. Edges represent significant Spearman correlations (| R| > 0.7, *P* < 0.05). Red lines indicate positive correlations, while green lines indicate negative correlations. **(B)** Network stability testing, where different colors represent different treatments, and the equation describes the change in natural connectivity. **(C)** Community complexity analysis, where different letters indicate statistically significant differences determined by *t*-test.

Co-occurrence networks were constructed based on Spearman correlation analysis to elucidate the effects of different ratios of the composite selenium activator on soil microbial community structure (Figures 5A, 6A). The results indicated that the treatments significantly affected both bacterial and fungal communities. Network stability was assessed by evaluating robustness via natural connectivity, and the results showed that the bacterial network stability of the Z5 treatment group was lower than that of other groups, while the Z8 treatment group exhibited higher network stability (Figures 5B, 6B). Similarly, in the fungal community, the Z4 treatment group showed lower network stability, whereas the Z8 treatment group again demonstrated higher stability. Furthermore, analysis of topological properties (Figures 5C, 6C) revealed that compared to the control group, only the Z5 and Z9 formulations increased the complexity of the bacterial network, but this was accompanied by reduced modularity. In contrast, the topological parameters of the fungal network generally showed a decreasing trend, indicating reduced network complexity but increased modularity. Furthermore, the network stability was assessed by evaluating the robustness via natural connectivity. The results showed that the Z5 group exhibited lower bacterial network stability than the other groups, while the Z8 treatment yielded a more stable network. Similarly, in the fungal community, the Z4 network showed lower stability, while the Z8 network again exhibited higher stability.

#### 3.3.4 Microbial community functional prediction analysis

Functional prediction analysis using PICRUSt2 was conducted to identify microbial functions influenced by the soil Se composite activator. Generally, the relative abundance of bacterial functional categories was similar across most samples in the ten treatment groups. Based on the KEGG database, five major metabolic pathways categories were identified: membrane transport, amino acid metabolism, cofactor and vitamin metabolism, energy metabolism, and nitrogen/sulfur metabolism. Further prediction at KEGG level 3 yielded 30 sub-functions (Supplementary Figure S1). Notably, the relative abundance of most predicted functions decreased in treatments where the composite activator was applied compared to the CK. The dominant functional categories included metabolic pathways, biosynthesis of secondary metabolites, microbial metabolism in diverse environments, biosynthesis of amino acids, carbon metabolism, ABC transporters, Two-component system, Ribosome, and Quorum sensing. Despite the general decrease, specific bacteria functions were significantly enhanced by the composite activator compared to CK, including aromatic compound degradation, ureolysis, nitrate reduction, and manganese oxidation. Furthermore, functions related to dark thiosulfate oxidation and dark oxidation of sulfur compounds were significantly enriched in treatments Z3, Z4, Z6, Z8, and Z9. Regarding fungi, FAPROTAX analysis revealed that the activator treatment reduced the relative abundance of saprotrophic groups (e.g., undefined saprotrophs, litter saprotrophs) and pathogen-saprotroph complexes, while concurrently increasing the abundance of plant pathogens.

### 3.4 Effects of microbial communities on soil properties and Se forms

**FIGURE 7:**
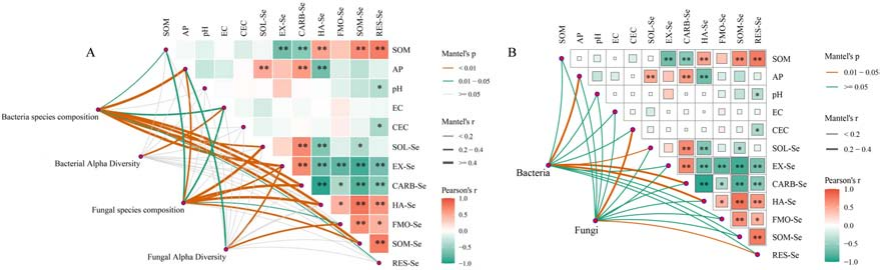
Correlation analysis between the microbial community and soil properties as well as Se species. **(A)** Correlation analysis between microbial community and diversity (at the genus level) with soil properties and Se species. **(B)** Correlation analysis between predicted microbial functions and soil properties as well as Se species. In **(A)**, orange, green, and grey lines denote positive correlations with *P* < 0.01, positive correlations with 0.05 > *P* ≥ 0.01, and non-significant correlations (*P* ≥ 0.05), respectively. In **(B)**, orange and green lines represent positive correlations (0.05 > *P* ≥ 0.01) and non-significant correlations (*P* ≥ 0.05), respectively. The width of each line corresponds to the Mantel’s r value, indicating the strength of the correlation. The heatmaps display Pearson correlation coefficients, with color intensity from green (negative) to orange (positive) representing the strength and direction of the linear relationship. Statistically significant correlations are marked with asterisks (**P < 0.01; *P < 0.05).

Figure 7A illustrates the correlation analysis among microbial community structure, diversity indices, soil properties, and Se fractions. The results indicate that microbial communities significantly influenced soil properties and Se speciation. Specifically, The structure of both bacterial and fungal communities exhibited strong, significant positive correlation with key soil properties (SOM, AP, EC) and Se fractions (SOL-Se, EX-Se, CARB-Se, HA-Se, SOM-Se). Regarding diversity, bacterial diversity was primarily influenced by EC, whereas fungal diversity was significantly correlated with AP, EX-Se, and FMO-Se. Notably, bioavailable Se content showed a significantly positive correlation with AP but a negative correlation with SOM. Consistent with the speciation analysis (Figure 2), where the activator increased CARB-Se while reducing HA-Se and SOM-Se, The bioavailable Se was significantly and positively correlated with bacterial and fungal community structures, as well as fungal diversity. Figure 7B depicts the correlations between predicted microbial functions and environmental variables. Bacterial functional profiles showed significant positive correlation with AP and specific Se fractions (SOL-Se, CARB-Se, HA-Se, SOM-Se). In contrast, fungal functional profiles were significantly and positively correlated with CEC and RES-Se.

## 4 Discussion

Before interpreting the specific mechanisms governing Se activation, it is crucial to acknowledge the experimental context of this study. The findings presented here are derived from strictly controlled laboratory incubation conditions with a limited soil mass (200 g) and an absence of active plant-soil interactions. Consequently, while the results illustrate the potential geochemical and microbial drivers of Se transformation, the direct quantitative extrapolation of these mechanisms to complex field environments has limitations and requires caution. Future work should involve systematic field trials to further evaluate the stability, economic feasibility, and long-term environmental behavior of the Se activator across different climates, soil types, and cropping systems, so as to facilitate its practical application. With these constraints in mind, the following discussion evaluates the proposed mechanisms based on the observed physicochemical shifts and microbial correlations.

### 4.1 The effect of compound Se activators on the soil Se forms

Se speciation in soil plays a critical role in governing its bioavailability. This study revealed that the application of a soil se activator complex significantly enhanced the concentrations of CARB-Se and the bioavailable se pool (comprising SOL-Se and EX-Se). Our data indicated that the Z8 treatment group exhibited the most significant effect, achieving a bioavailable Se content of 39.2 μg/kg (a 56% increase).

The superior performance of Z8, characterized by a lower dosage of PT, suggests a synergistic interaction between its phosphorus component and organic matter. Consistent with previous literature, we hypothesized that Phosphorus activates Se by competing for adsorption sites on soil colloids and by increasing soil pH—mechanisms that theoretically promote the transformation of stable Se fractions into CARB-Se and SOL-Se (Fan et al., 2018). Furthermore, the superior performance of Z8 compared to Z2 is likely associated with its higher concentration of WV, As source of low-molecular-weight organic acids (Fang et al., 2019), WV may exert a competitive-synergistic coupling effect that reduces Se(IV) adsorption. Although not directly measured in this study, these acids are known to activate Se by potentially degrading clay minerals (Zhang M. et al., 2019) and dissociating organic-Se complexes via chelation. Such processes would facilitate the release of SOL-Se and EX-Se for subsequent recombination with carbonate minerals.

Significantly, the activator treatment reduced the proportions of HA-Se and SOM-Se. This aligns with the hypothesis that the higher concentration of BFA in Z8 (compared to Z2) contribute to the degradation of organic complexes and the release of bound Se (Yang et al., 2024). In contrast, the highly stable fractions (FMO-Se and RES-Se) remained largely unaffected, indicating that the activation strategy preferentially targets labile and semi-labile Se pools, with limited efficacy on recalcitrant fractions fixed within crystal lattices (Khan et al., 2017).

### 4.2 Effect of Se composite activator on basic soil properties

The results indicate that the application of composite activators can regulate soil physicochemical properties, likely through multiple synergistic pathways. Soil SOM content exhibited a biphasic trend: an initial slow increase followed by accelerated accumulation. This pattern is consistent with a priming effect often induced by exogenous organic matter inputs (Nottingham et al., 2009; Chen et al., 2014). Additionally, the colloidal adsorption capacity of BFA and AA likely improves soil structure and enhances aggregate stability, thereby promotes SOM retention (Liu et al., 2023). Concurrently, a significant increase in soil pH was observed. This shift is plausibly linked to SOM mineralization, a process generating organic ligands that undergo exchange reactions with hydrous iron oxides and aluminum hydroxides, thereby consuming H^+^ (Dijkstra and Keitel, 2024). BFA may further mitigate acidification by directly complexing or adsorbing protons (Liu et al., 2023). Moreover, the dissolution of silicates and calcium/magnesium ions from PT could provide a neutralizing effect, contributing to the pH elevation (Buss et al., 2024). Regarding Available Phosphorus (AP), the observed increase over time suggests a mechanism involving the acid dissolution of phosphate from phosphorus residues by organic acids (Bai et al., 2024). Importantly, the enhancement of AP may be mechanistically coupled with Se release through competitive adsorption. Since phosphorus is preferentially adsorbed onto iron oxide surfaces, it potentially displaces Se into the solution, thereby increasing Se bioavailability (Duc et al., 2003; Huang et al., 2024).

### 4.3 The effect of compound Se activators on the microbial communities and diversity in the soil

The application of the Z8 compound Se activator significantly altered the microbial community structure. These shifts in bacterial and fungal abundance were strongly correlated with changes in Se speciation, suggesting that biological mechanisms, such as reduction and methylation, may complement chemical activation in enhancing Se bioavailability.

Specifically, the Z8 treatment significantly enriched *Arthrobacter* and *Bacillus*, while reducing *Sphingomonas*. Genera such as *Bacillus* are widely recognized in the literature for their roles in Se metabolism (Oremland et al., 2004). Although metabolic rates were not quantified here, the enrichment of these taxa implies a potential for converting soluble selenite (SeO_3_^2–^) and selenate (SeO_4_^2–^) into insoluble elemental Se (Se^0^) (Ahmed et al., 2024). If active, this pathway would reduce Se toxicity and facilitate immobilization, potentially increasing bioavailability via the formation of biogenic Se nanoparticles (SeNPs). Similarly, the increased abundance of Arthrobacter supports the hypothesis of biological Se reduction, as this genus is capable of converting Se(IV) to Se^0^ (Nie et al., 2023). The presence of *Lysinibacillus* further suggests a potential contribution to biomineralization processes influencing Se solubility (Oremland et al., 2004).

In the fungal community, the activator increased the abundance of genera functionally associated with Se reduction and methylation, including Fusarium, Trichoderma, and Aspergillus. Fusarium has been reported to convert inorganic Se into volatile organic compounds. Furthermore, Trichoderma and Aspergillus have been implicated in SeNP biosynthesis in previous studies, processes that could enhance Se availability (Ojeda et al., 2020; Arshad et al., 2021). While these microbial shifts point toward beneficial plant-microbe interactions—such as the potential recruitment of plant growth-promoting rhizobacteria (PGPRs) noted in other systems—it is important to reiterate that these implications remain hypothetical within the current incubation setup (Babajide et al., 2025). Future field-scale trials involving vegetation are essential to validate whether these specific microbial community changes translate into improved crop Se uptake and agronomic performance.

## 5 Conclusion

This study demonstrates that a novel soil Se activator, composed of organic acids and PT at an optimal ratio (30% BFA, 10% alginate, 50% WV, and 20% PT), significantly (*P* < 0.05) increased the content of SOL-Se and EX-Se in soil without exogenous supplementation. Mechanistically, the activator promoted the transformation of HA-Se and SOM-Se into more labile forms (CARB-Se and SOL-Se + EX-Se), in soil without exogenous Se supplementation. The activator also promoted the transformation of HA-Se and SOM-Se into CARB-Se and SOL-Se + EX-Se, thereby enhancing soil Se bioavailability. Furthermore, the treatment significantly increased soil AP content and effectively regulated soil pH, These findings provide a theoretical basis for the resource utilization of industrial PT waste and the activation of native soil Se.

## Data Availability

The datasets presented in this study can be found in online repositories. The names of the repository/repositories and accession number(s) can be found in the article/Supplementary material.

## References

[B1] Ahmed F. Zhang D. Tang X. Malakar P. K. (2024). Targeting spore-forming bacteria: A review on the antimicrobial potential of selenium nanoparticles. *Foods* 13:4026. 10.3390/foods13244026 39766969 PMC11728422

[B2] Arshad H. Sami M. A. Sadaf S. Hassan U. (2021). Salvadora persica mediated synthesis of silver nanoparticles and their antimicrobial efficacy. *Sci. Rep.* 11:5996. 10.1038/s41598-021-85584-w 33727607 PMC7966387

[B3] Babajide A. M. Adebami G. E. Adebayo-Tayo B. C. (2025). Screening of rhizobacteria from monkey pod trees for plant growth promoters and evaluating the antifungal potential of the biosynthesized selenium nanoparticles. *Sci. Rep.* 15:16797. 10.1038/s41598-025-96330-x 40369107 PMC12078570

[B4] Bai Y. Weng L. Hiemstra T. (2024). Interaction of fulvic acid with soil organo-mineral nano-aggregates and corresponding phosphate release. *Geoderma* 441:116737. 10.1016/j.geoderma.2023.116737

[B5] Buss W. Hasemer H. Ferguson S. Borevitz J. (2024). Stabilisation of soil organic matter with rock dust partially counteracted by plants. *Glob. Chang Biol.* 30:e17052. 10.1111/gcb.17052 37994295

[B6] Chen L. Zhang M. Liu D. Sun H. Wu J. Huo Y. et al. (2022). Designing specific bacterial 16S primers to sequence and quantitate plant endo-bacteriome. *Sci. China Life Sci.* 65 1000–1013. 10.1007/s11427-021-1953-5 34309738

[B7] Chen R. Senbayram M. Blagodatsky S. Myachina O. Dittert K. Lin X. et al. (2014). Soil C and N availability determine the priming effect: Microbial N mining and stoichiometric decomposition theories. *Glob. Chang Biol.* 20 2356–2367. 10.1111/gcb.12475 24273056

[B8] Chen S. Zhou Y. Chen Y. Gu J. (2018). fastp: An ultra-fast all-in-one FASTQ preprocessor. *Bioinformatics* 34 i884–i890. 10.1093/bioinformatics/bty560 30423086 PMC6129281

[B9] Cheng H. Zhang D. Ren L. Song Z. Li Q. Wu J. et al. (2021). Bio-activation of soil with beneficial microbes after soil fumigation reduces soil-borne pathogens and increases tomato yield. *Environ. Pollut.* 283:117160. 10.1016/j.envpol.2021.117160 33878684

[B10] de la Mora M. L. Pinilla L. Rosas A. Cartes P. (2008). Selenium uptake and its influence on the antioxidative system of white clover as affected by lime and phosphorus fertilization. *Plant Soil* 303 139–149. 10.1007/s11104-007-9494-z

[B11] Deng G. Fan Z. Wang Z. Peng M. (2025). Dynamic role of selenium in soil–plant-microbe systems: Mechanisms, biofortification, and environmental remediation. *Plant Soil* 515 1085–1105. 10.1007/s11104-025-07661-7

[B12] Di Tullo P. Pannier F. Thiry Y. Le Hécho I. Bueno M. (2016). Field study of time-dependent selenium partitioning in soils using isotopically enriched stable selenite tracer. *Sci. Total Environ.* 562 280–288. 10.1016/j.scitotenv.2016.03.207 27100008

[B13] Dijkstra F. A. Keitel C. (2024). Maximising carbon sequestration through mixing compost in moist soil. *Soil Biol. Biochem.* 191:109330. 10.1016/j.soilbio.2024.109330

[B14] Duc M. Lefevre G. Fedoroff M. Jeanjean J. Rouchaud J. C. Monteil-Rivera F. et al. (2003). Sorption of selenium anionic species on apatites and iron oxides from aqueous solutions. *J. Environ. Radioact.* 70 61–72. 10.1016/S0265-931X(03)00125-5 12915060

[B15] Eswayah A. S. Smith T. J. Gardiner P. H. (2016). Microbial transformations of selenium species of relevance to bioremediation. *Appl. Environ. Microbiol.* 82 4848–4859. 10.1128/AEM.00877-16 27260359 PMC4968552

[B16] Fan J. Zeng Y. Sun J. (2018). The transformation and migration of selenium in soil under different Eh conditions. *J. Soils Sediments* 18 2935–2947. 10.1007/s11368-018-1980-9

[B17] Fan Q. Fan X. Fu P. Li Y. Zhao Y. Hua D. (2022). Anaerobic digestion of wood vinegar wastewater using domesticated sludge: Focusing on the relationship between organic degradation and microbial communities (archaea, bacteria, and fungi). *Bioresour Technol.* 347:126384. 10.1016/j.biortech.2021.126384 34808316

[B18] Fang D. Wei S. Xu Y. Xiong J. Tan W. (2019). Impact of low-molecular weight organic acids on selenite immobilization by goethite: Understanding a competitive-synergistic coupling effect and speciation transformation. *Sci. Total Environ.* 684 694–704. 10.1016/j.scitotenv.2019.05.294 31174097

[B19] Fellowes J. W. Pattrick R. A. D. Boothman C. Al Lawati W. M. M. van Dongen B. E. Charnock J. M. et al. (2013). Microbial selenium transformations in seleniferous soils. *Eur. J. Soil Sci.* 64 629–638. 10.1111/ejss.12051

[B20] Fischer S. Krause T. Lederer F. Merroun M. L. Shevchenko A. Hübner R. et al. (2020). Bacillus safensis JG-B5T affects the fate of selenium by extracellular production of colloidally less stable selenium nanoparticles. *J. Hazard Mater*. 384:121146. 10.1016/j.jhazmat.2019.121146 31771888

[B21] Gong H. Zhai H. Wang Y. Pan L. Liu Y. Zhang Y. et al. (2024). Changes in selenium bioavailability in selenium-enriched paddy soils induced by different water management and organic amendments. *Sci. Total Environ.* 957:177844. 10.1016/j.scitotenv.2024.177844 39631343

[B22] Han L. Chen Y. Chen M. Wu Y. Su R. Du L. et al. (2020). Mushroom residue modification enhances phytoremediation potential of Paulownia fortunei to lead-zinc slag. *Chemosphere* 253:126774. 10.1016/j.chemosphere.2020.126774 32464764

[B23] Huang J. Huang X. Jiang D. (2024). Phosphorus can effectively reduce selenium adsorption in selenium-rich lateritic red soil. *Sci. Total Environ.* 906:167356. 10.1016/j.scitotenv.2023.167356 37769720

[B24] Khalid M. U. Imran M. Aslam M. Ashraf M. (2022). The interactive effect of selenium and farmyard manure on soil microbial activities, yield and selenium accumulation by wheat (*Triticum aestivum* L.) grains. *J. Plant Growth Regul.* 41 2669–2677. 10.1007/s00344-021-10465-5

[B25] Khan A. M. Bakar N. K. A. Bakar A. F. A. Ashraf M. A. (2017). Chemical speciation and bioavailability of rare earth elements (REEs) in the ecosystem: A review. *Environ. Sci. Pollut. Res. Int.* 24 22764–22789. 10.1007/s11356-016-7427-1 27722986

[B26] Kushwaha A. Goswami L. Lee J. Sonne C. Brown R. J. C. Kim K.-H. (2022). Selenium in soil-microbe-plant systems: Sources, distribution, toxicity, tolerance, and detoxification. *Crit. Rev. Environ. Sci. Technol.* 52 2383–2420. 10.1080/10643389.2021.1883187

[B27] Li J. Awasthi M. K. Xing W. Liu R. Bao H. Wang X. et al. (2020). Arbuscular mycorrhizal fungi increase the bioavailability and wheat (*Triticum aestivum* L.) uptake of selenium in soil. *Ind. Crops Prod.* 150:112383. 10.1016/j.indcrop.2020.112383

[B28] Li Z. Liang D. Peng Q. Cui Z. Huang J. Lin Z. (2017). Interaction between selenium and soil organic matter and its impact on soil selenium bioavailability: A review. *Geoderma* 295 69–79. 10.1016/j.geoderma.2017.02.019

[B29] Liao Q. Xing Y. Li A.-M. Liang P.-X. Jiang Z.-P. Liu Y.-X. et al. (2024). Enhancing selenium biofortification: Strategies for improving soil-to-plant transfer. *Chem. Biol. Technol. Agric.* 11:148. 10.1186/s40538-024-00672-z

[B30] Liu M. Tan X. Zheng M. Yu D. Lin A. Liu J. et al. (2023). Modified biochar/humic substance/fertiliser compound soil conditioner for highly efficient improvement of soil fertility and heavy metals remediation in acidic soils. *J. Environ. Manage.* 325(Pt A):116614. 10.1016/j.jenvman.2022.116614 36419293

[B31] Liu N. Wang M. Zhou F. Zhai H. Qi M. Liu Y. et al. (2021). Selenium bioavailability in soil-wheat system and its dominant influential factors: A field study in Shaanxi province. China. *Sci. Total Environ.* 770:144664. 10.1016/j.scitotenv.2020.144664 33513517

[B32] Liu Q. Chen Z. Tang J. Luo J. Huang F. Wang P. et al. (2022). Cd and Pb immobilisation with iron oxide/lignin composite and the bacterial community response in soil. *Sci. Total Environ.* 802:149922. 10.1016/j.scitotenv.2021.149922 34525730

[B33] Mora M. L. Durán P. Acuña J. Cartes P. Demanet R. Gianfreda L. (2015). Improving selenium status in plant nutrition and quality. *J. Soil Sci. Plant Nutr.* 15 486–503. 10.4067/S0718-95162015005000041 27315006

[B34] Moreno-Jiménez E. Clemente R. Mestrot A. Meharg A. A. (2013). Arsenic and selenium mobilisation from organic matter treated mine spoil with and without inorganic fertilisation. *Environ. Pollut.* 173 238–244. 10.1016/j.envpol.2012.10.017 23202981

[B35] Najafi-Ghiri M. Mirsoleimani A. Boostani H. R. Amin H. (2022). Influence of wood vinegar and potassium application on soil properties and Ca/K ratio in citrus rootstocks. *J. Soil Sci. Plant Nutr.* 22 334–344. 10.1007/s42729-021-00653-3

[B36] Nie X. Yang X. He J. Liu P. Shi H. Wang T. et al. (2023). Bioconversion of inorganic selenium to less toxic selenium forms by microbes: A review. *Front. Bioeng. Biotechnol.* 11:1167123. 10.3389/fbioe.2023.1167123 36994362 PMC10042385

[B37] Nottingham A. T. Griffiths H. Chamberlain P. M. Stott A. W. Tanner E. V. J. (2009). Soil priming by sugar and leaf-litter substrates: A link to microbial groups. *Appl. Soil Ecol.* 42 183–190. 10.1016/j.apsoil.2009.03.003

[B38] Ojeda J. J. Merroun M. L. Tugarova A. V. Lampis S. Kamnev A. A. Gardiner P. H. E. (2020). Developments in the study and applications of bacterial transformations of selenium species. *Crit. Rev. Biotechnol.* 40 1250–1264. 10.1080/07388551.2020.1811199 32854560

[B39] Oremland R. S. Herbel M. J. Blum J. S. Langley S. Beveridge T. J. Ajayan P. M. et al. (2004). Structural and spectral features of selenium nanospheres produced by Se-respiring bacteria. *Appl. Environ. Microbiol.* 70 52–60. 10.1128/AEM.70.1.52-60.2004 14711625 PMC321302

[B40] Peng Q. Wang M. Cui Z. Huang J. Chen C. Guo L. et al. (2017). Assessment of bioavailability of selenium in different plant-soil systems by diffusive gradients in thin-films (DGT). *Environ. Pollut.* 225 637–643. 10.1016/j.envpol.2017.03.036 28341328

[B41] Pinel-Cabello M. Chapon V. Ruiz-Fresneda M. A. Alpha-Bazin B. Berthomieu C. Armengaud J. et al. (2021). Delineation of cellular stages and identification of key proteins for reduction and biotransformation of Se(IV) by *Stenotrophomonas bentonitica* BII-R7. *J. Hazard. Mater.* 418:126150. 10.1016/j.jhazmat.2021.126150 34111750

[B42] Qi M. Wang D. Zhai H. Zhou F. Wu H. Zhao W. et al. (2025). Effects of straw amendment on the bioavailability of selenite in soil and its mechanisms. *Ecotoxicol. Environ. Saf.* 290:117578. 10.1016/j.ecoenv.2024.117578 39709708

[B43] Qiao J. Li X. Li F. Liu T. Young L. Y. Huang W. et al. (2019). Humic substances facilitate arsenic reduction and release in flooded paddy soil. *Environ. Sci. Technol.* 53 5034–5042. 10.1021/acs.est.8b06333 30942579

[B44] Qu L. Xu J. Dai Z. Elyamine A. M. Huang W. Han D. et al. (2023). Selenium in soil-plant system: Transport, detoxification and bioremediation. *J. Hazard Mater.* 452:131272. 10.1016/j.jhazmat.2023.131272 37003006

[B45] Ros G. H. van Rotterdam A. M. D. Bussink D. W. Bindraban P. S. (2016). Selenium fertilization strategies for bio-fortification of food: An agro-ecosystem approach. *Plant Soil.* 404 99–112. 10.1007/s11104-016-2830-4

[B46] Stackebrandt E. Goebel B. M. (1994). Taxonomic note: A place for DNA-DNA reassociation and 16S rRNA sequence analysis in the present species definition in bacteriology. *Int. J. Syst. Evol. Microbiol.* 44 846–849. 10.1099/00207713-44-4-846

[B47] Supriatin S. Terrones C. A. Bussink W. Weng L. (2015). Drying effects on selenium and copper in 0.01 M calcium chloride soil extractions. *Geoderma* 25 104–114. 10.1016/j.geoderma.2015.04.021

[B48] Urík M. Bujdoš M. Gardošová K. Littera P. Matúš P. (2018). Selenite distribution in multicomponent system consisting of filamentous fungus, humic acids, bentonite, and ferric oxyhydroxides. *Water Air Soil Pollut.* 229, 1–7. 10.1007/s11270-018-3719-z

[B49] Wang D. Dinh Q. T. Anh Thu T. T. Zhou F. Yang W. Wang M. et al. (2018). Effect of selenium-enriched organic material amendment on selenium fraction transformation and bioavailability in soil. *Chemosphere* 199 417–426. 10.1016/j.chemosphere.2018.02.007 29453068

[B50] Wang D. Rensing C. Zheng S. (2022). Microbial reduction and resistance to selenium: Mechanisms, applications and prospects. *J. Hazard Mater.* 421:126684. 10.1016/j.jhazmat.2021.126684 34339989

[B51] Wang D. Xue M. Y. Wang Y. K. Zhou D. Z. Tang L. Cao S. Y. et al. (2019). Effects of straw amendment on selenium aging in soils: Mechanism and influential factors. *Sci. Total Environ.* 657 871–881. 10.1016/j.scitotenv.2018.12.021 30677952

[B52] Wang G. Li Z. Yang B. Yang H. Zhang Y. Zeng Q. et al. (2024). The effect of white grub (Maladera Verticalis) larvae feeding on rhizosphere microbial characterization of aerobic rice (*Oryza sativa* L.) in Puer City, Yunnan Province, China. *BMC Microbiol.* 24:123. 10.1186/s12866-024-03265-w 38622504 PMC11017655

[B53] Wang Q. Garrity G. M. Tiedje J. M. Cole J. R. (2007). Naive Bayesian classifier for rapid assignment of rRNA sequences into the new bacterial taxonomy. *Appl. Environ. Microbiol.* 73 5261–5267. 10.1128/AEM.00062-07 17586664 PMC1950982

[B54] Wang Z. Huang W. Pang F. (2022). Selenium in soil-plant-microbe: A review. *Bull. Environ. Contam. Toxicol.* 108 167–181. 10.1007/s00128-021-03386-2 34617141

[B55] Wen Y. C. Li H. Y. Lin Z. A. Zhao B. Q. Sun Z. B. Yuan L. et al. (2020). Long-term fertilization alters soil properties and fungal community composition in fluvo-aquic soil of the North China Plain. *Sci. Rep.* 10:7198. 10.1038/s41598-020-64227-6 32350351 PMC7190697

[B56] Wu F. Ren Y. Qu G. Liu S. Chen B. Liu X. et al. (2022). Utilization path of bulk industrial solid waste: A review on the multi-directional resource utilization path of phosphogypsum. *J. Environ. Manage.* 313:114957. 10.1016/j.jenvman.2022.114957 35390656

[B57] Xing M. Cao Q. Wang Y. Xiao H. Zhao J. Zhang Q. et al. (2020). Advances in research on the bioactivity of alginate oligosaccharides. *Mar. Drugs* 18:144. 10.3390/md18030144 32121067 PMC7142810

[B58] Xu Z. N. Lin Z. Q. Zhao G. S. Guo Y. B. (2024). Biogeochemical behavior of selenium in soil-air-water environment and its effects on human health. *Int. J. Environ. Sci. Technol.* 21 1159–1180. 10.1007/s13762-023-05169-0

[B59] Yang J.-S. Xie Y.-J. He W. (2011). Research progress on chemical modification of alginate: A review. *Carbohydr. Polym.* 84 33–39. 10.1016/j.carbpol.2010.11.048

[B60] Yang X. Mao K. Chang C. Wang J. Wu Q. Shao Y. et al. (2024). Interactions of selenium and mercury in soil–plant systems: Characterizations, occurrences, and mechanisms. *Crit. Rev. Environ. Sci. Technol.* 54 1527–1545. 10.1080/10643389.2024.2332135

[B61] Zhang F. Shao J. Yang H. Guo D. Chen Z. Zhang S. et al. (2019). Effects of biomass pyrolysis derived wood vinegar on microbial activity and communities of activated sludge. *Bioresour. Technol.* 279 252–261. 10.1016/j.biortech.2019.01.133 30735935

[B62] Zhang M. Xing G. Tang S. Pang Y. Yi Q. Huang Q. et al. (2019). Improving soil selenium availability as a strategy to promote selenium uptake by high-Se rice cultivar. *Environ. Exp. Bot.* 163 45–54. 10.1016/j.envexpbot.2019.04.008

[B63] Zheng L. Bian W. Jiang J. Wang P. Li Y. Cui Q. et al. (2025). Wood vinegar promotes dissolved organic matter transformation in rice straw: Mechanistic insights into fulvic acid uptake and nitrogen enrichment. *J. Environ. Chem. Eng.* 13:119743. 10.1016/j.jece.2025.119743

[B64] Zhou Z. Yan T. Zhu Q. Bu X. Chen B. Xue J. et al. (2019). Bacterial community structure shifts induced by biochar amendment to karst calcareous soil in southwestern areas of China. *J. Soils Sediments* 19 356–365. 10.1007/s11368-018-2035-y

