# Supplementary material for: Soil selenium activator mediates selenium form transformation and affects soil properties and microbial communities
Source: Front Microbiol. 2026 Apr 15;17:1715630. doi: 10.3389/fmicb.2026.1715630 (PMC13127118; doi:10.3389/fmicb.2026.1715630)
Supplement: Supplementary file 1 [file Data_Sheet_1.docx]

**Table 1.** The main components of wood vinegar consist of a blended liquid derived from grape branches, corn stalks, fir wood, and miscellaneous wood, with specific compositional details provided in the table below.

| Grape branch | English name | Molecular formula | Retention time (min) | Comparative content(%) |
| --- | --- | --- | --- | --- |
| Acids | Lactic acid | C_3_H_6_O_3_ | 7.42 | 11.59 |
|  | 2-ketoadipate | C_6_H_8_O_5_ | 10.05 | 9.48 |
|  | Picolinic acid | C_6_H_5_NO_2_ | 11.09 | 9.4 |
|  | 2-hydroxybutanoic acid | C_4_H_8_O_3_ | 8.33 | 6.35 |
|  | Glycolic acid | C_2_H_4_O_3_ | 7.68 | 4.31 |
|  | Stearic acid | C_18_H_36_O_2_ | 21.11 | 2.97 |
|  | N-Ethylglycine | C_4_H_9_NO_2_ | 12.42 | 2.88 |
|  | Adipic acid | C_6_H_10_O_4_ | 13.38 | 1.31 |
|  | Palmitic acid | C_16_H_32_O_2_ | 19.32 | 1.15 |
|  | Others | —— | —— | 2.3 |
| Phenols | Guaiacol | C_7_H_8_O_2_ | 9.84 | 8.48 |
|  | Catechol | C_6_H_6_O_2_ | 11.06 | 7.36 |
|  | M-cresol | C_7_H_8_O | 8.63 | 5.01 |
|  | O-cresol | C_7_H_8_O | 8.5 | 1.33 |
|  | Others | —— | —— | 0.17 |
| Ketones | 1,3-Cyclohexanedione | C_6_H_8_O_2_ | 10.74 | 3.15 |
|  | 1,2-Cyclohexanedione | C_6_H_8_O_2_ | 15.33 | 1.47 |
|  | Others | —— | —— | 0.16 |
| Esters | D-erythronolactone | C_4_H_6_O_4_ | 12.47 | 1.09 |
|  | Linoleic acid methyl ester | C_19_H_34_O_2_ | 19.77 | 1 |
|  | Others | —— | —— | 0.12 |
| Aldehydes | Glutaraldehyde | C_5_H_8_O_2_ | 10.54 | 1.18 |
|  | Succinate semialdehyde | C_4_H_6_O_3_ | 9.2 | 1.13 |
|  | 2,5-dihydroxybenzaldehyde | C_7_H_6_O_3_ | 15.94 | 1.08 |
|  | Others | —— | —— | 0.08 |
| Alcohols | Perillyl Alcohol | C_10_H_16_O | 12.21 | 1.45 |
|  | 3-Methylbenzyl Alcohol | C_8_H_10_O | 10 | 0.48 |
|  | (-)-Dihydrocarveol | C_10_H_18_O | 10.72 | 0.36 |
|  | Acetol | C_3_H_6_O_2_ | 13.71 | 0.27 |
|  | 3-Methylamino-1,2-propanediol | C_4_H_11_NO_2_ | 9.46 | 0.26 |
|  | Others | —— | —— | 0.19 |
| Unknown | —— | —— | —— | 12.52 |

| Corn stalks | English name | Molecular formula | Retention time(min) | Comparative content(%) |  |
| --- | --- | --- | --- | --- | --- |
|  |  |  |  |  |  |
| Acids | N-Ethylglycine | C_4_H_9_NO_2_ | 12.42 | 7.98 |  |
|  | 2-ketoadipate | C_6_H_8_O_5_ | 10.05 | 6.94 |  |
|  | Lactic acid | C_3_H_6_O_3_ | 7.42 | 2.78 |  |
|  | 2-hydroxybutanoic acid | C_4_H_8_O_3_ | 8.33 | 1.96 |  |
|  | Palmitic acid | C_16_H_32_O_2_ | 19.32 | 1.9 |  |
|  | Picolinic acid | C_6_H_5_NO_2_ | 11.09 | 1.75 |  |
|  | Glycolic acid | C_2_H_4_O_3_ | 7.68 | 1.67 |  |
|  | Stearic acid | C_18_H_36_O_2_ | 21.11 | 1.66 |  |
|  | Adipic acid | C_6_H_10_O_4_ | 13.38 | 1.56 |  |
|  | Others | —— | —— | 4.9 |  |
| Phenols | Catechol | C_6_H_6_O_2_ | 11.06 | 5.51 |  |
|  | Guaiacol | C_7_H_8_O_2_ | 9.84 | 3.37 |  |
|  | M-cresol | C_7_H_8_O | 8.63 | 2.53 |  |
|  | O-cresol | C_7_H_8_O | 8.5 | 1.29 |  |
|  | Others | —— | —— | 0.17 |  |
| Ketones | 1,3-Cyclohexanedione | C_6_H_8_O_2_ | 10.74 | 5.58 |  |
|  | 1,2-Cyclohexanedione | C_6_H_8_O_2_ | 15.33 | 1.71 |  |
|  | Others | —— | —— | 0.23 |  |
| Esters | D-erythronolactone | C_4_H_6_O_4_ | 12.47 | 3.12 |  |
|  | Linoleic acid methyl ester | C_19_H_34_O_2_ | 19.77 | 1.01 |  |
|  | Others | —— | —— | 0.15 |  |
| Aldehydes | Glutaraldehyde | C_5_H_8_O_2_ | 10.54 | 10.22 |  |
|  | Succinate semialdehyde | C_4_H_6_O_3_ | 9.2 | 4.21 |  |
|  | Others | —— | —— | 0.06 |  |
| Alcohols | Perillyl Alcohol | C_10_H_16_O | 12.21 | 9.4 |  |
|  | 3-Methylbenzyl Alcohol | C_8_H_10_O | 10 | 1.6 |  |
|  | 3-Methylamino-1,2-propanediol | C_4_H_11_NO_2_ | 9.46 | 0.51 |  |
|  | (+)-Dihydrocarveol | C_10_H_18_O | 10.72 | 0.36 |  |
|  | Acetol | C_3_H_6_O_2_ | 13.71 | 0.21 |  |
|  | Others | —— | —— | 0.22 |  |
| Unknown | —— | —— | —— | 15.44 |  |

| Fir wood | English name | Molecular formula | Retention time/min | Comparative content(%) |  |
| --- | --- | --- | --- | --- | --- |
|  |  |  |  |  |  |
| Acids | palmitic acid | C_16_H_32_O_2_ | 19.32 | 12.93 |  |
|  | lactic acid | C_3_H_6_O_3_ | 7.42 | 8.73 |  |
|  | 2-picolinic acid | C_6_H_5_NO_2_ | 11.09 | 5.68 |  |
|  | 2-ketoadipate | C_6_H_8_O_5_ | 10.05 | 4.56 |  |
|  | N-Ethylglycine | C_4_H_9_NO_2_ | 12.42 | 4.49 |  |
|  | adipic acid | C_6_H_10_O_4_ | 13.38 | 2.99 |  |
|  | stearic acid | C_18_H_36_O_2_ | 21.11 | 2.98 |  |
|  | glycolic acid | C_2_H_4_O_3_ | 7.68 | 1.77 |  |
|  | 2-hydroxybutanoic acid | C_4_H_8_O_3_ | 8.33 | 0.64 |  |
|  | Others |  | —— | 1.6 |  |
| phenols | guaiacol | C_7_H_8_O_2_ | 9.84 | 11.39 |  |
|  | catechol | C_6_H_6_O_2_ | 11.06 | 9.32 |  |
|  | m-cresol | C_7_H_8_O | 8.63 | 2.82 |  |
|  | o-cresol | C_7_H_8_O | 8.5 | 1.29 |  |
|  | Others |  | —— | 0.14 |  |
| Ketones | 1,3-Cyclohexanedione | C_6_H_8_O_2_ | 10.74 | 3.56 |  |
|  | 1,2-Cyclohexanedione | C_6_H_8_O_2_ | 15.33 | 2.38 |  |
|  | Others |  | —— | 0.13 |  |
| Esters | D-erythronolactone | C_4_H_6_O_4_ | 12.47 | 3.14 |  |
|  | Linoleic acid methyl ester | C_19_H_34_O_2_ | 19.77 | 1.01 |  |
|  | Others |  | —— | 0.09 |  |
| Aldehydes | glutaraldehyde | C_5_H_8_O_2_ | 10.54 | 1.23 |  |
|  | 2,5-dihydroxybenzaldehyde | C_7_H_6_O_3_ | 15.94 | 1.11 |  |
|  | succinate semialdehyde | C_4_H_6_O_3_ | 9.2 | 1.1 |  |
|  | Others |  | —— | 0.06 |  |
| Alcohols | Perillyl Alcohol | C_10_H_16_O | 12.21 | 3.54 |  |
|  | 3-Methylbenzyl Alcohol | C_8_H_10_O | 10 | 0.58 |  |
|  | (+)-Dihydrocarveol | C_10_H_18_O | 10.72 | 0.44 |  |
|  | 3-Methylamino-1,2-propanediol | C_4_H_11_NO_2_ | 9.46 | 0.29 |  |
|  | Acetol | C_3_H_6_O_2_ | 13.71 | 0.29 |  |
|  | Others |  | —— | 0.16 |  |
| Unknown | —— |  | —— | 10.78 |  |

| Miscellaneous wood | English name | Molecular formula | Retention time/min | Comparative content(%) |
| --- | --- | --- | --- | --- |
| Acids | N-Ethylglycine | C_4_H_9_NO_2_ | 12.42 | 18.46 |
|  | lactic acid | C_3_H_6_O_3_ | 7.42 | 14.3 |
|  | 2-picolinic acid | C_6_H_5_NO_2_ | 11.09 | 10.25 |
|  | palmitic acid | C_16_H_32_O_2_ | 19.32 | 4.9 |
|  | 2-ketoadipate | C_6_H_8_O_5_ | 10.05 | 3.46 |
|  | glycolic acid | C_2_H_4_O_3_ | 7.68 | 2 |
|  | stearic acid | C_18_H_36_O_2_ | 21.11 | 1.64 |
|  | adipic acid | C_6_H_10_O_4_ | 13.38 | 1.37 |
|  | Others |  | —— | 0.9 |
| phenols | catechol | C_6_H_6_O_2_ | 11.06 | 13.14 |
|  | guaiacol | C_7_H_8_O_2_ | 9.84 | 11.04 |
|  | m-cresol | C_7_H_8_O | 8.63 | 1.64 |
|  | o-cresol | C7H8O | 8.5 | 0.34 |
|  | Others |  | —— | 0.29 |
| Ketones | 1,3-Cyclohexanedione | C_6_H_8_O_2_ | 10.74 | 1.03 |
|  | 1,2-Cyclohexanedione | C_6_H_8_O_2_ | 15.33 | 1.03 |
|  | Others |  | —— | 0.41 |
| Esters | Methyl jasmonate | C_13_H_20_O_3_ | 16.17 | 1.19 |
|  | D-erythronolactone | C4H6O4 | 12.47 | 0.1 |
|  | Others |  | —— | 0.04 |
| Aldehydes | succinate semialdehyde | C_4_H_6_O_3_ | 9.2 | 3.25 |
|  | glutaraldehyde | C_5_H_8_O_2_ | 10.54 | 0.2 |
|  | 2,5-dihydroxybenzaldehyde | C_7_H_6_O_3_ | 15.94 | 0.17 |
|  | butyraldehyde | C_4_H_8_O | 10.5 | 0.15 |
|  | Others |  | —— | 0.03 |
| Alcohols | Perillyl Alcohol | C_10_H_16_O | 12.21 | 1.96 |
|  | 3-Methylamino-1,2-propanediol | C_4_H_11_NO_2_ | 9.46 | 0.44 |
|  | (-)-Dihydrocarveol | C_10_H_18_O | 10.72 | 0.29 |
|  | 3-Methylbenzyl Alcohol | C_8_H_10_O | 10 | 0.13 |
|  | Acetol | C_3_H_6_O_2_ | 13.71 | 0.12 |
|  | Others |  | —— | 0.26 |
| Unknown | —— |  | —— | 5.66 |

**Table 2.** Composition of Alginate Compound Fertilizer

| Composition | Organic Matter | Alginic Acid | Ca | Mg | B | Zn | Cu | Fe | Mn |
| --- | --- | --- | --- | --- | --- | --- | --- | --- | --- |
| Content/% | 30 | 12 | 0.7 | 0.3 | 1 | 0.7 | 0.2 | 0.15 | 0.05 |

Composition of Bio-fulvic Acid Fertilizer: 70% Bio-fulvic Acid, 30% Organic Matter.

**Table 3.** Composition of Phosphorus Tailings：

| Composition | P_2_O_5_ | MgO | SiO_2_ | CaO | CO_2_ | SO_2_ | K_2_O | F | Fe_2_O_3_ | LOI |
| --- | --- | --- | --- | --- | --- | --- | --- | --- | --- | --- |
| Content/% | 8.63 | 15.24 | 5.35 | 34.14 | 24.53 | 5.14 | 2.23 | 0.37 | 0.86 | 3.47 |

Risk Assessment

Based on the compositional analysis of the phosphorus tailings, the potential risks associated with their application to soil are as follows:

The phosphorus tailings contain fluorine. However, the fluorine content of 0.37% is generally below the common fluorine limit standards (typically below 0.5%-1.0%). Under reasonable and controlled application rates, the risks of soil fluorine accumulation and phytotoxicity become manageable.

The phosphorus tailings contain a significant amount of carbonates (e.g., CaCO₃, MgCO₃), which are alkaline. Direct application into the soil could cause soil alkalization. Therefore, we have combined them with organic acids such as fulvic acid, wood vinegar, and seaweed acid for co-application. This combination plays a role in acid-base neutralization, thus allowing the alkalizing effect of the phosphorus tailings on the soil to be neglected.

The Fe₂O₃ content in the phosphorus tailings has low toxicity, and its level of 0.86% is negligible.

Therefore, under the combined application of this phosphorus tailings with organic acids, the risk of harm to the soil is relatively low, and it can be used in appropriate quantities.


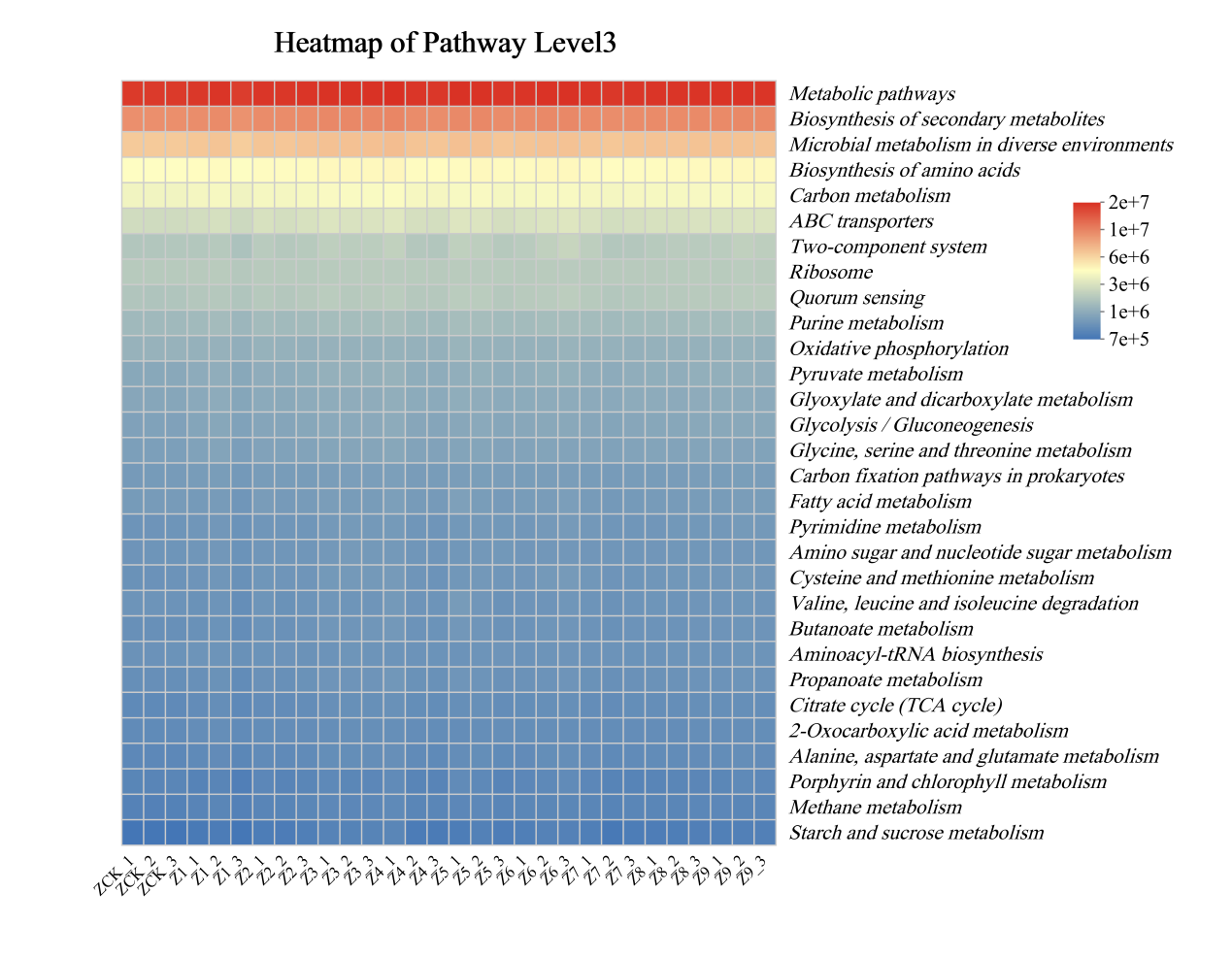


Fig 1. Heatmap of bacterial metabolic pathways at Level 3.


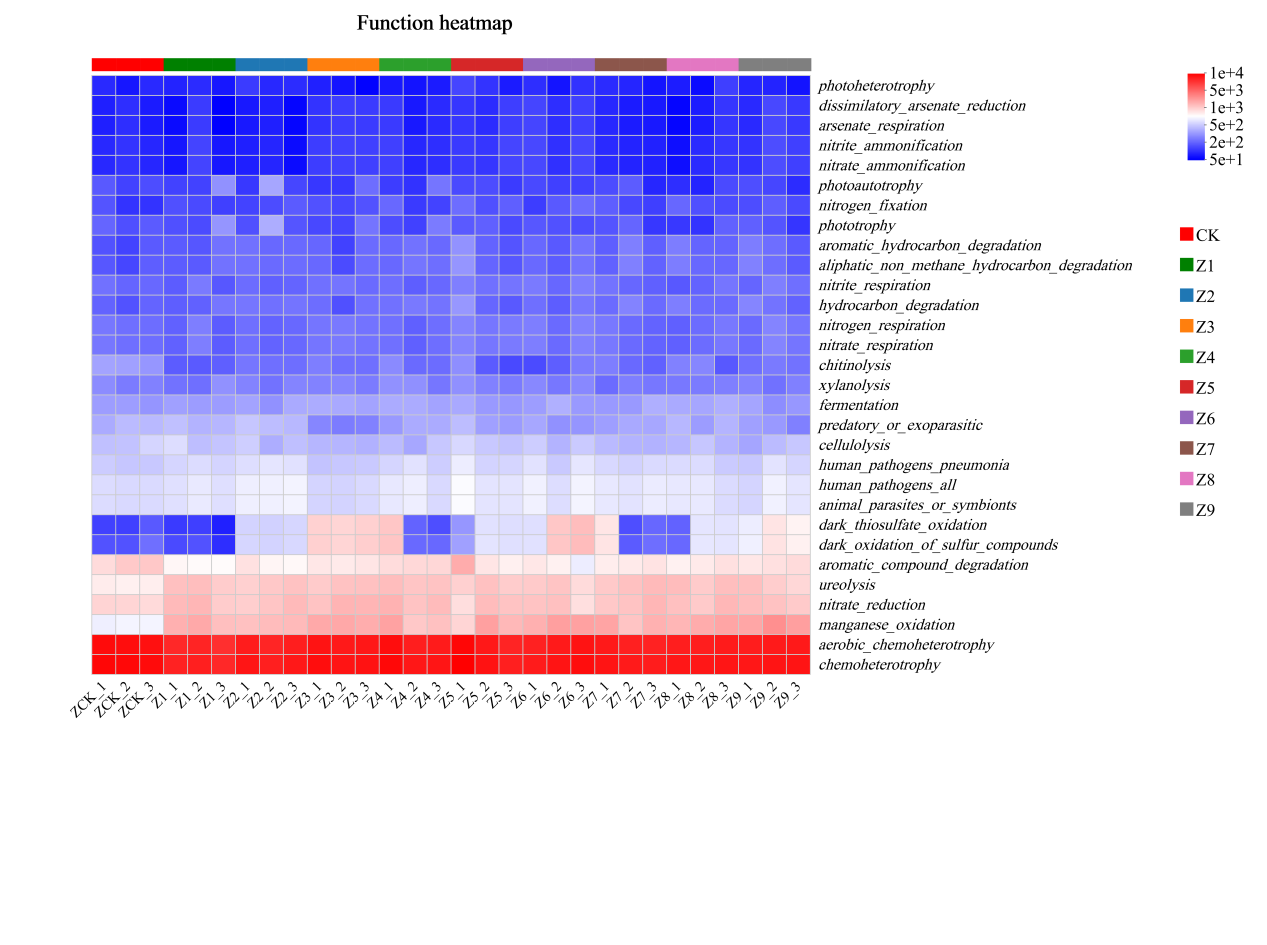


Fig 2. Functional heatmap of bacteria.


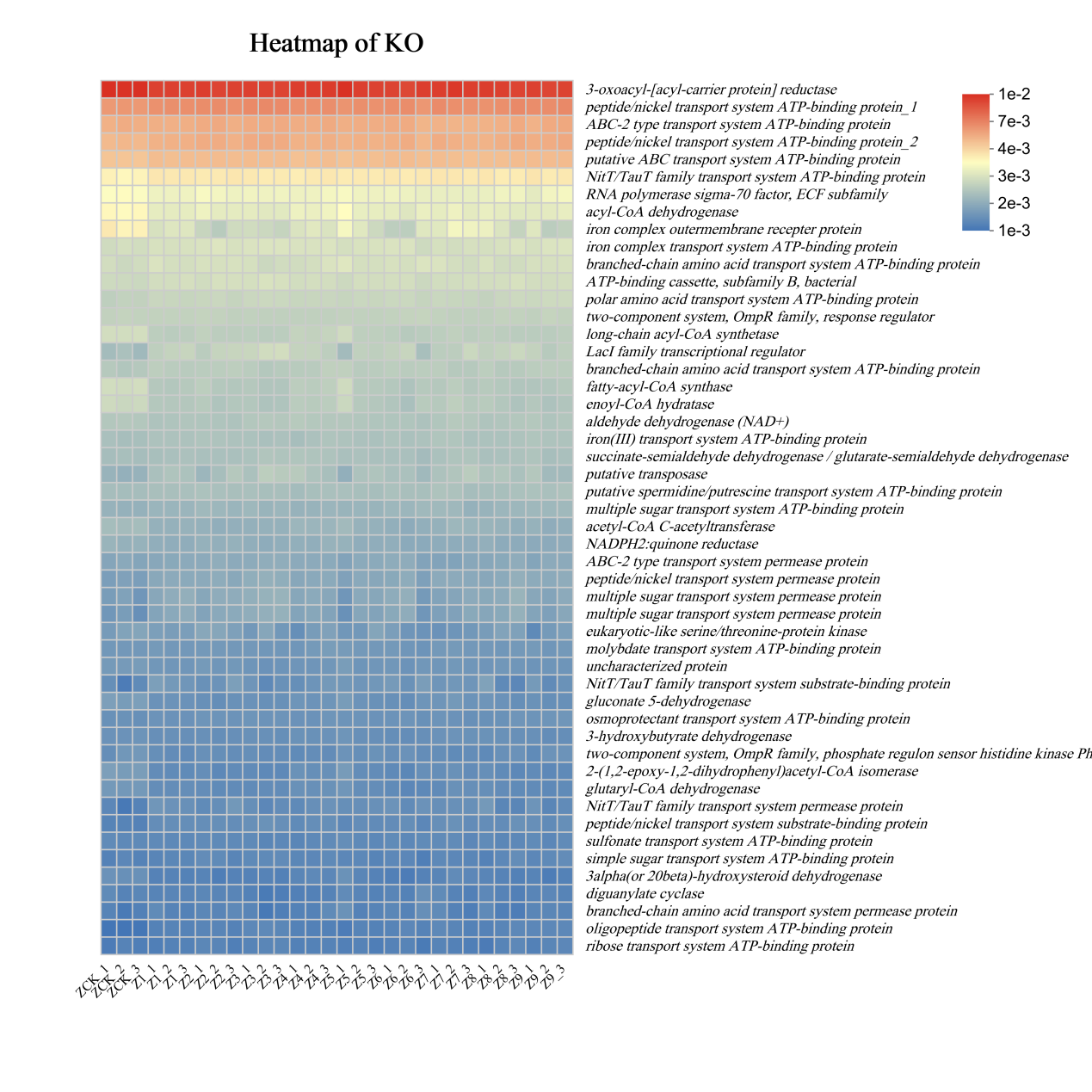


Fig 3. Heatmap of bacterial KEGG pathways (Reference database: Ref100NR).


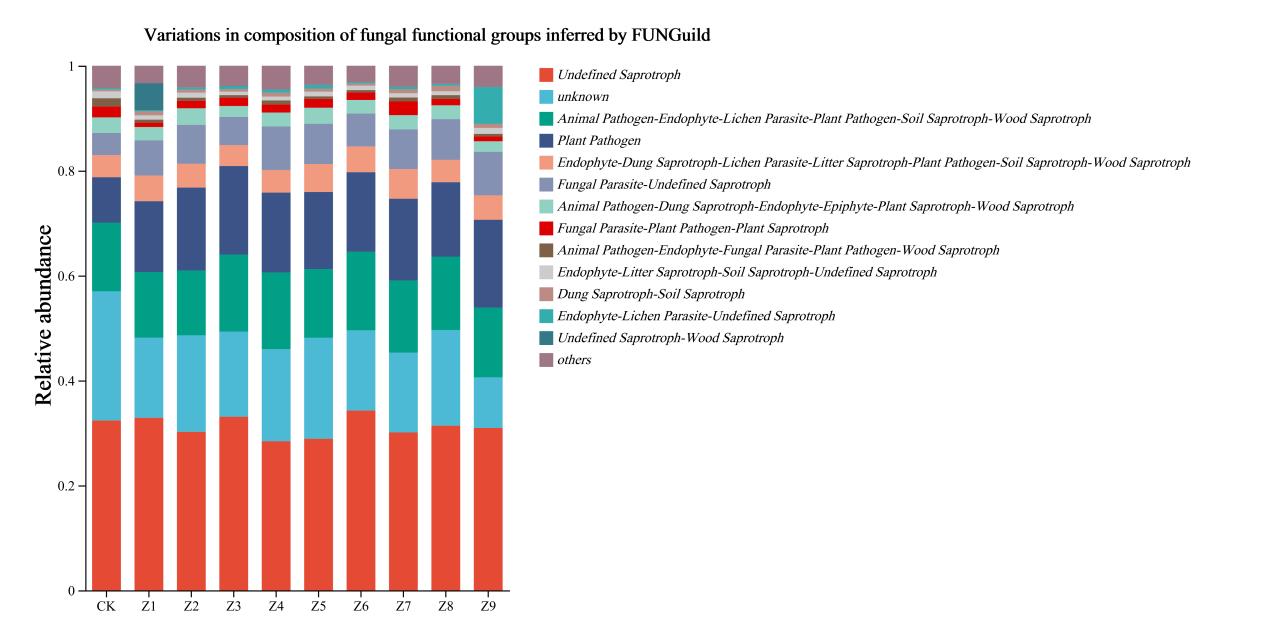


Fig 4. Variations in composition of fungal functional groups predicted by FUNGuild.

**Table 4** Specific Microbial Functional Genera in Z8 vs CK Treatments.

| serial  number | CK endemic bacteria | Z8 endemic bacteria | CK endemic fungi | Z8 endemic fungi |
| --- | --- | --- | --- | --- |
| 1 | *g__A0839* | *01D2Z36* | *g__Arthrocladium* | g__Acidomelania |
| 2 | *g__AB1* | *028H05-P-BN-P5* | *g__Badarisama* | g__Albonectria |
| 3 | *g__Acidicaldus* | *37-13* | *g__Bovista* | g__Apiotrichum |
| 4 | *g__Acidisphaera* | *AT-s3-44* | *g__Bradymyces* | g__Aplosporella |
| 5 | *g__Asticcacaulis* | *Acidimicrobiaceae* | *g__Calcarisporiella* | g__Arthrographis |
| 6 | *g__BD1-7_clade* | *Acidiphilium* | *g__Collarina* | g__Candida |
| 7 | *g__Balneolaceae* | *Acuticoccus* | *g__Coniochaeta* | g__Chaetosphaeria |
| 8 | *g__Beggiatoaceae* | *Alkaliphilus* | *g__Coprinopsis* | g__Coprinellus |
| 9 | *g__Berkelbacteria* | *Alterococcus* | *g__Cordyceps* | g__Cutaneotrichosporon |
| 10 | *g__Bordetella* | *Anaerolinea* | *g__Corynascella* | g__Emmonsiellopsis |
| 11 | *g__Bradymonadales* | *Anaeromyxobacter* | *g__Didymella* | g__Hannaella |
| 12 | *g__Brockia* | *Babeliaceae* | *g__Disciseda* | g__Hypoxylon |
| 13 | *g__C1-B045* | *CM1G08* | *g__Exserohilum* | g__Inocybe |
| 14 | *g__Caedibacter* | *Candidatus_Jorgensenbacteria* | *g__Iodophanus* | g__Inonotus |
| 15 | *g__Camelimonas* | *Candidatus_Koribacter* | *g__Keissleriella* | g__Lasiodiplodia |
| 16 | *g__Candidatus_Bealeia* | *Candidatus_Levybacteria* | *g__Leucothecium* | g__Lecanicillium |
| 17 | *g__Candidatus_Captivus* | *Candidatus_Nucleicultrix* | *g__Lophotrichus* | g__Leptoxyphium |
| 18 | *g__Candidatus_Megaira* | *Candidatus_Xiphinematobacter* | *g__Loweporus* | g__Octospora |
| 19 | *g__Candidatus_Metachlamydia* | *Cellulosilyticum* | *g__Mariannaea* | g__Paracremonium |
| 20 | *g__Caulobacteraceae* | *Chroococcidiopsis_PCC_7203* | *g__Melanospora* | g__Pluteus |
| 21 | *g__Cavicella* | *Chthonomonadales* | *g__Microthecium* | g__Pseudopithomyces |
| 22 | *g__Chungangia* | *Dadabacteriales* | *g__Monocillium* | g__Trichosporon |
| 23 | *g__Cyanobacteriales* | *Elev-1554* | *g__Papiliotrema* | g__Veronaeopsis |
| 24 | *g__Cytophaga* | *FFCH16767* | *g__Paramicrothyrium* | g__Vishniacozyma |
| 25 | *g__D8A-2* | *Ferrovibrionales* | *g__Perenniporia* | g__unclassified_c__Eurotiomycetes |
| 26 | *g__Desmonostoc_PCC-7422* | *Frankiales* | *g__Pestalotiopsis* | g__unclassified_f__Lophiostomataceae |
| 27 | *g__Desulfitibacter* | *G12-WMSP1* | *g__Phaeosphaeria* | g__unclassified_f__Orbiliaceae |
| 28 | *g__Desulfitobacteriales* | *Gracilibacter* | *g__Podospora* | g__unclassified_f__Piptocephalidaceae |
| 29 | *g__EF100-94H03* | *Hahella* | *g__Preussia* | g__unclassified_o__Helotiales |
| 30 | *g__Edaphobacter* | *Ideonella* | *g__Ramichloridium* |  |
| 31 | *g__Elsteraceae* | *Inquilinus* | *g__Rectifusarium* |  |
| 32 | *g__Emticicia* | *Jan-59* | *g__Sarocladium* |  |
| 33 | *g__Estrella* | *Kouleothrix* | *g__Serpula* |  |
| 34 | *g__Euzebya* | *Ktedonobacterales* | *g__Setophoma* |  |
| 35 | *g__Flindersiella* | *Lachnoclostridium* | *g__Simocybe* |  |
| 36 | *g__Fluviicola* | *MM2* | *g__Sphaeropsis* |  |
| 37 | *g__Geobacillus* | *Modestobacter* | *g__Spiromastix* |  |
| 38 | *g__Geothermomicrobium* | *Moorella* | *g__Sporormiella* |  |
| 39 | *g__Granulicella* | *Neisseriaceae* | *g__Sporothrix* |  |
| 40 | *g__HN-HF0106* | *Olivibacter* | *g__Stachybotrys* |  |
| 41 | *g__Herbinix* | *Paucimonas* | *g__Stagonosporopsis* |  |
| 42 | *g__Izemoplasmatales* | *Propionicicella* | *g__Thanatephorus* |  |
| 43 | *g__Kibdelosporangium* | *Romboutsia* | *g__Ustilaginoidea* |  |
| 44 | *g__Kiloniellaceae* | *Subgroup_11* | *g__Yuchengia* |  |
| 45 | *g__Ktedonobacter* | *Subgroup_2* | *g__Yunnania* |  |
| 46 | *g__Lacibacter* | *Sulfobacillaceae* | *g__unclassified_c__Dothideomycetes* |  |
| 47 | *g__Lacunisphaera* | *WWE3* | *g__unclassified_f__Clavicipitaceae* |  |
| 48 | *g__Leptolyngbyaceae* | *norank* | *g__unclassified_f__Dictyosporiaceae* |  |
| 49 | *g__Lutispora* | *unclassified_Arenicellaceae* | *g__unclassified_f__Dipodascaceae* |  |
| 50 | *g__Methylovirgula* | *unclassified_Chlamydiaceae* | *g__unclassified_f__Glomeraceae* |  |
| 51 | *g__Microcoleus_SAG_1449-1a* | *unclassified_Clostridiaceae* | *g__unclassified_f__Sordariaceae* |  |
| 52 | *g__Nitrosococcus* | *unclassified_Ilumatobacteraceae* | *g__unclassified_f__Spizellomycetaceae* |  |
| 53 | *g__Opitutaceae* | *unclassified_Patescibacteria* | *g__unclassified_f__Stephanosporaceae* |  |
| 54 | *g__Oryzihumus* | *unclassified_Phycisphaeraceae* | *g__unclassified_o__Branch03* |  |
| 55 | *g__PHOS-HE36* | *unclassified_Rhodobacteraceae* | *g__unclassified_o__Capnodiales* |  |
| 56 | *g__Paracaedibacteraceae* | *unclassified_Steroidobacteraceae* | *g__unclassified_o__Glomerellales* |  |
| 57 | *g__Parafilimonas* | *unclassified_Symbiobacteraceae* | *g__unclassified_o__Trechisporales* |  |
| 58 | *g__Phormidium_IAM_M-71* |  | *g__unclassified_p__Glomeromycota* |  |
| 59 | *g__Pir4_lineage* |  |  |  |
| 60 | *g__Pla4_lineage* |  |  |  |
| 61 | *g__Planosporangium* |  |  |  |
| 62 | *g__Polyangium* |  |  |  |
| 63 | *g__Pseudoduganella* |  |  |  |
| 64 | *g__Pullulanibacillus* |  |  |  |
| 65 | *g__Puniceispirillales* |  |  |  |
| 66 | *g__Roseococcus* |  |  |  |
| 67 | *g__Ruminococcus* |  |  |  |
| 68 | *g__Simkaniaceae* |  |  |  |
| 69 | *g__Smaragdicoccus* |  |  |  |
| 70 | *g__Sphingorhabdus* |  |  |  |
| 71 | *g__Streptosporangium* |  |  |  |
| 72 | *g__Subgroup_13* |  |  |  |
| 73 | *g__Synechococcus_IR11* |  |  |  |
| 74 | *g__Taonella* |  |  |  |
| 75 | *g__Tessaracoccus* |  |  |  |
| 76 | *g__Thermaerobacter* |  |  |  |
| 77 | *g__Thermithiobacillus* |  |  |  |
| 78 | *g__Thermoleophilia* |  |  |  |
| 79 | *g__UCG-010* |  |  |  |
| 80 | *g__Undibacterium* |  |  |  |
| 81 | *g__VHS-B3-70* |  |  |  |
| 82 | *g__Verrucomicrobiaceae* |  |  |  |
| 83 | *g__Virgibacillus* |  |  |  |
| 84 | *g__unclassified_Acidobacteriota* |  |  |  |
| 85 | *g__unclassified_Anaerolineae* |  |  |  |
| 86 | *g__unclassified_Caloramatoraceae* |  |  |  |
| 87 | *g__unclassified_Chitinophagaceae* |  |  |  |
| 88 | *g__unclassified_Chthoniobacteraceae* |  |  |  |
| 89 | *g__unclassified_Diplorickettsiaceae* |  |  |  |
| 90 | *g__unclassified_Hyphomicrobiaceae* |  |  |  |
| 91 | *g__unclassified_Ktedonobacteria* |  |  |  |
| 92 | *g__unclassified_Nostocaceae* |  |  |  |
| 93 | *g__unclassified_Parcubacteria* |  |  |  |
| 94 | *g__unclassified_Planctomycetota* |  |  |  |
| 95 | *g__unclassified_Polyangiales* |  |  |  |
| 96 | *g__unclassified_Proteobacteria* |  |  |  |
| 97 | *g__unclassified_Rickettsiales* |  |  |  |
| 98 | *g__unclassified_Thermoleophilia* |  |  |  |
| 99 | *g__unclassified_Vicinamibacterales* |  |  |  |
| 100 | *g__unclassified_Xanthomonadales* |  |  |  |

The revised draft has been polished by professional English editing institutions. Specific as follows
